# National, regional, and provincial prevalence of age-related macular degeneration in China in 2020: an updated systematic review and modelling study

**DOI:** 10.7189/jogh.16.04062

**Published:** 2026-01-23

**Authors:** Jing Wu, Shiyi Shan, Jiali Zhou, Yanqing Li, Qianqian Ke, Longzhu Zhu, Igor Rudan, Peige Song, Global Health Epidemiology Research Group (GHERG)

**Affiliations:** 1 Center for Clinical Big Data and Statistics of the Second Affiliated Hospital Zhejiang University School of Medicine, School of Public Health Zhejiang University School of Medicine, Hangzhou, Zhejiang, China; 2 Zhejiang Key Laboratory of Intelligent Preventive Medicine, Hangzhou, Zhejiang, China; 3 Department of Infectious Diseases and Public Health, City University of Hong Kong, Hong Kong, China; 4 School of Public Health, Cheeloo College of Medicine, Shandong University, Jinan, Shandong, China; 5 Global Business School for Health, Faculty of Population Health Sciences, University College London, London, UK; 6 Centre for Global Health, Usher Institute, University of Edinburgh, Edinburgh, Scotland, UK; 7 Nuffield Department of Primary Care Health Sciences, Oxford University, Oxford, UK

## Abstract

**Background:**

The burden of age-related macular degeneration (AMD) has steadily increased in recent decades. We aimed to estimate the prevalence of AMD, including its subtypes, among individuals aged 40–89 years in China.

**Methods:**

We conducted an updated literature search in the CNKI, Wanfang, Chinese Science and Technology Journal Database, PubMed, Embase, and MEDLINE for studies published between 27 June 2016 and 30 July 2024 that reported on the prevalence of AMD in China. We also included data from the 2017 China AMD Study. We utilised a multi-level mixed-effects meta-regression model to estimate age- and sex-specific prevalence of any AMD and its subtypes at the national level. For any AMD, we additionally conducted random-effects meta-analyses to pool odds ratios for associated factors, after which we incorporated these estimates into an associated factor-based model to estimate prevalence at regional and provincial levels.

**Results:**

We included 40 articles, of which 24 contributed data for modelling analysis. The estimated national prevalence in China in 2020 was 4.70% (95% CI = 3.40, 6.46) for any AMD, 4.06% (95% CI = 2.92, 5.60) for early AMD, and 0.64% (95% CI = 0.48, 0.86) for late AMD, including 0.30% (95% CI = 0.25, 0.37) for geographic atrophy and 0.34% (95% CI = 0.23, 0.49) for neovascular AMD. These corresponded to 32.42 million cases (95% CI = 23.43, 44.54) with any AMD, 28.00 million (95% CI = 20.15, 38.61) with early AMD, 4.42 million (95% CI = 3.28, 5.93) with late AMD, 2.09 million (95% CI = 1.71, 2.52) with geographic atrophy, and 2.33 million (95% CI = 1.57, 3.41) with neovascular AMD. Regionally, the highest prevalence and number of cases was observed in Southwest China (5.95%; 95% CI = 4.48, 7.81) and South Central China (10.68 million; 95% CI = 7.60, 14.82), respectively. At the provincial level, Hainan and Guangdong exhibited the highest prevalence (7.64%; 95% CI = 4.61, 12.22) and the largest number of individuals affected (3.50 million; 95% CI = 2.34, 5.13), respectively.

**Conclusions:**

We observed a substantial burden of AMD in Mainland China, with variations across subtypes, regions, and provinces. These findings underscore a need for targeted public health strategies to address AMD in the context of ageing.

**Registration:**

PROSPERO: CRD420251080685.

Age-related macular degeneration (AMD), a progressive degenerative disease of the central retina, is one of the leading causes of irreversible visual impairment and blindness among older adults worldwide [1]. It is clinically classified by stage of progression into early or late AMD, with the former often manifesting as asymptomatic, and the latter (comprising neovascular AMD (NVAMD) and geographic atrophy (GA)) causing central vision loss and impairing essential activities such as reading, driving, and recognising faces [2,3]. Beyond vision loss, AMD is associated with increased risks of depression, falls, loss of independence, and reduced quality of life across multiple domains, placing a substantial burden on individuals, caregivers, and health systems [4–7].

The global burden of AMD has increased steadily over recent decades – a trend largely driven by population ageing [8]. China, which accounts for nearly one-fifth of the global population, is undergoing one of the most rapid demographic transitions in the world. In 2017, the Global Health Epidemiology and Research Group (GHERG) conducted the first modelling study of AMD in the country (the 2017 China AMD Study), providing national and regional prevalence estimates from 1990 to 2015, with projections to 2050 [9].

Emerging evidence has highlighted that AMD prevalence is shaped not only by age and disease subtype, but also by geographic and sociodemographic factors, including urbanisation, latitude, income, education, and modifiable exposures such as smoking, diet, and metabolic disorders [2,10,11]. These determinants vary considerably across China, giving rise to substantial heterogeneity in AMD prevalence between provinces [12]. In this context, understanding the provincial distribution of AMD is essential to ensure equity in service provision, guide resource allocation, and inform targeted prevention strategies.

Despite its public health importance, conducting a national survey for AMD that would encompass all provinces of China remains logistically and financially prohibitive. In such settings, model-based approaches that synthesise data from population-based studies offer a practical and scalable alternative. However, estimates from the 2017 China AMD Study, which took on such an approach, have been limited to national or broad regional levels [9]. Since its publication, several new epidemiological studies have been conducted in China [13,14], expanding the evidence base and enabling refinement of existing models.

Here, we aimed to address this data gap through an updated systematic review and statistical modelling. Using a global health metrics approach developed by GHERG, we estimated the prevalence of any AMD and its subtypes (early AMD, late AMD, GA, and NVAMD) in Mainland China in 2020 at national, regional, and provincial levels. Specifically, we wanted to update the national prevalence of any AMD, early AMD, late AMD, GA, and NVAMD; estimate the regional prevalence of any AMD across various regions; and generate the first-ever provincial-level estimates of any AMD prevalence.

## METHODS

The protocol for this review was registered in PROSPERO (CRD420251080685). We report our findings in accordance with the PRISMA guidelines and the GATHER statement [15,16]. 

### Search strategy and selection criteria

Building on our 2017 China AMD Study [9], we performed an updated literature search in three Chinese (CNKI, Wanfang Data, Chinese Science and Technology Journal Database) and three global (PubMed, Embase, MEDLINE) databases to identify population-based studies published from 27 June 2016 to 30 July 2024 reporting the prevalence of AMD in the general population of China. The search strategy used terms related to AMD (*e.g.* ‘age-related macular degeneration’, ‘age-related maculopathy’), prevalence (*e.g.* ‘prevalence’, ‘epidemiology’, ‘morbidity’), and China (applied only in English databases), with adaptation to each databases (Appendix 1 in the **Online Supplementary Document**). We also screened the reference lists of all included articles to identify additional eligible studies.

Following deduplication, two investigators (YL and QK) independently screened titles and abstracts of all retrieved records, followed by the full text of any that they deemed potentially eligible. We included population-based epidemiological investigations conducted in China that reported numerical estimates of AMD prevalence or provided sufficient data for calculation (*i.e.* sample size and number of cases). The estimated AMD prevalence had to be based on the number of affected individuals, rather than the number of affected eyes. The studies were also required to use clearly defined assessment methods, diagnostic criteria, and grading systems, with those based solely on self-reported diagnoses not being considered for inclusion. We further excluded non-original studies (*e.g. *reviews, editorials, conference abstracts, case reports, letters, and viewpoints) and studies that focused on unrepresentative populations, such as individuals with visual impairment or other underlying diseases (*e.g.* diabetes). For multiple publications reporting on the same investigation, we retained the one with the most detailed results or the largest sample size.

During the systematic review process, we observed that the studies used different AMD grading systems, such as the ‘Age-related Macular Degeneration Clinical Diagnosis Standard’ proposed by the China Medical Association in 1986 [17], the International Classification and Grading system [18], the Clinical Age-Related Maculopathy Grading System [19], the grading system proposed by the Age-Related Eye Disease Study Research Group [20], the Wisconsin age-related maculopathy system [21], or the Beckmann classification system [22]. To maximise comparability and enable data synthesis across heterogeneous studies, we included only those that reported on either any AMD or its standard clinical subtypes (*i.e.* early AMD, late AMD, GA, and NVAMD). Specifically, late AMD was consistently defined across all systems as the presence of GA or NVAMD. For early AMD, definitions varied slightly regarding drusen size thresholds. To address this, we adopted a harmonised definition covering all early-stage variations. Where studies reported intermediate AMD as a separate category, we classified them under early AMD to align with systems utilising broader classifications.

### Data extraction and quality assessment

Two investigators (YL and QK) independently extracted the following data using a standardised data extraction form, with checks from a third investigator (JW):

− study characteristics, including first author, publication year, investigation year, study location, study setting (mixed, urban, rural), study design, sampling method, AMD assessment method, and AMD grading system;

− population characteristics, including sample size, proportion of female participants, and age (reported as mean, median, or range);

− prevalence estimates, including the number of AMD cases, stratified by age group (lower and upper bounds), sex, setting, and AMD subtype, where available.

We also extracted the geographic characteristics of each study location, determining its latitude and longitude using Google Maps GPS coordinates [23], and its altitude and annual insolation through the NASA POWER Data Access Viewer [24]. For studies that reported broad geographical units (*e.g.* provinces or multi-city regions), we assigned geographic coordinates based on the mean centre point of the area. Then, we categorised each study location into one of six major geographical regions in Mainland China (North China, Northeast China, East China, South Central China, Southwest China, and Northwest China) and one of four economic regions (East, Central, West, and Northeast) (Table S1 in the **Online Supplementary Document**).

For studies where the investigation year was not reported, we imputed the year as three years prior to publication, based on the average lag observed in studies where both dates were available (Table S2 in the **Online Supplementary Document**). In cases where the lower or upper boundary of an age group was censored, we imputed the missing value using one of two approaches: if the mean age of the group was available, we calculated the boundary as a weighted average; otherwise, we assigned the missing age band the same width as adjacent age categories within the same study.

For a subset of studies that reported associated factors of any AMD using multivariable logistic regression, we additionally extracted the definition of each factor, along with the adjusted odds ratios (ORs) and corresponding 95% confidence intervals (CIs).

We assessed the methodological quality of each study using the Joanna Briggs Institute critical appraisal tool (Table S3 in the **Online Supplementary Document**) [25]. The tool comprises nine questions, with the answer ‘yes’ scored 1, and the answers ‘no’, ‘unclear’, and ‘not applicable’ scored 0. The total scores range from 0 to 9 and indicate the overall risk of bias, whereby scores 0–3 indicate low quality, 4–6 indicate moderate quality, and 7–9 indicate high quality studies [26,27].

Any discrepancies during screening, data extraction, and quality assessment were resolved by discussion or, if consensus was not reached, through by consulting a senior researcher (PS).

### Statistical analysis

**Figure 1:**
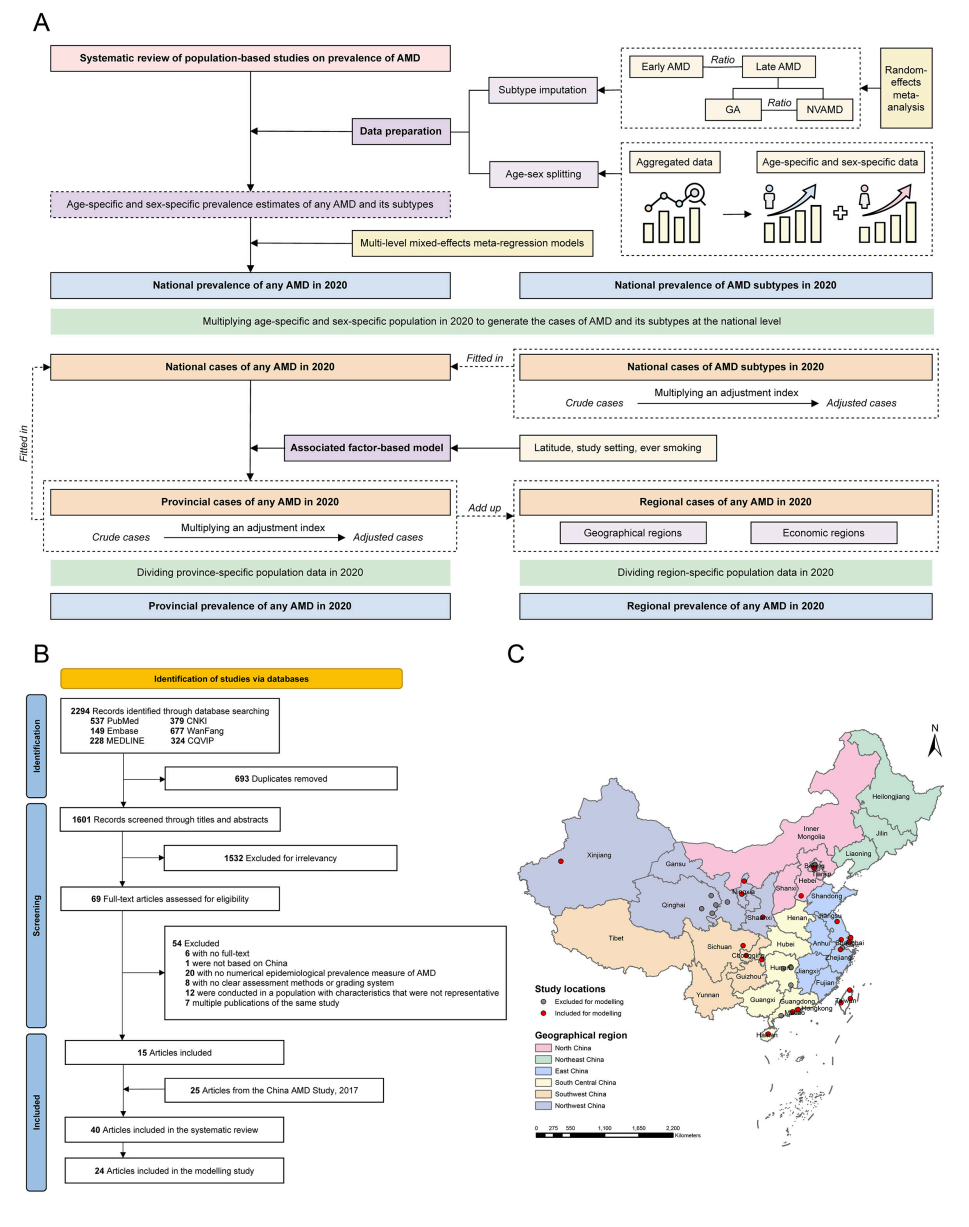
Study flowchart and geographical distribution of included studies. **Panel A.** Study approach. **Panel B.** PRISMA flow diagram of included articles. **Panel C.** Geographical distribution of included studies. AMD – age-related macular degeneration. GA – geographic atrophy, NVAMD – neovascular AMD.

We conducted a four-stage modelling analysis to estimate the national, regional, and provincial prevalence of any AMD or its subtypes in China in 2020 (**Figure 1**, Panel A): data preparation, including AMD subtype imputation and age-sex splitting (stage 1); estimation of national prevalence of any AMD and its four subtypes (early AMD, late AMD, GA, and NVAMD) in 2020 (stage 2); estimation of the effects of factors associated with any AMD (stage 3); estimation of regional and provincial prevalence of any AMD in 2020 (stage 4). The analysis included only population-based studies conducted in or after 1990 and assessed as high quality (defined as quality score ≥7). To ensure reliability and comparability across studies and subtypes, we further restricted it to individuals aged 40–89 years, which covered most of the data points available for modelling.

#### Data preparation

To maximise the use of available data, we imputed missing subtype information using pooled ratios. Specifically, we estimated the ratio of early to late AMD among any AMD, and the ratio of GA to NVAMD among late AMD, using random-effects meta-analysis based on the DerSimonian and Laird method (Figures S1–4 and Table S4 in the **Online Supplementary Document**). We then applied these pooled ratios to disaggregate any AMD prevalence into subtype-specific estimates when subtype data were not directly reported. For studies that reported age- or sex-aggregated prevalence, we applied a robust age-sex splitting procedure to derive age-specific and sex-specific prevalence estimates for any AMD and all subtypes (Appendix 2 in the **Online Supplementary Document**).

#### National prevalence estimation of any AMD and its subtypes in 2020

We used multi-level mixed-effects meta-regression models to estimate the age-specific and sex-specific prevalence of any AMD and its subtypes at the national level. The models included fixed effects for age and sex (female proportion) and random intercepts for study and province identifiers to account for clustering. The model was fitted using a logit transformation of prevalence:



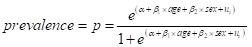



Here, *α* is the intercept term, *β* is the fixed-effect coefficient, and *u_i_* is the study-level random-effect.

We then applied the modelled age-sex-specific prevalence estimates to population data from the 2020 China national census to calculate the estimated number of individuals aged 40–89 years living with any AMD or its subtypes [28]. An ‘adjustment index’ was used to ensure internal consistency between the total number of any AMD cases and the sum of subtype-specific case estimates. We performed sensitivity analyses for any AMD by additionally including studies with quality scores <7 or investigation year before 1990.

#### Estimation of the effects of factors associated with any AMD

We assessed the association between any AMD prevalence and study-level covariates using a series of separate multivariable meta-regression models. We adjusted each model for age and sex, with one additional covariate added individually to assess its effect (Table S5 in the **Online Supplementary Document**). Covariates included investigation year, publication year, geographic coordinates (latitude and longitude), altitude, solar insolation, and study setting (urban *vs*. rural). For associated factors reported in at least three studies using multivariable logistic regression (Table S6 in the **Online Supplementary Document**), we conducted random-effects meta-analysis to pool adjusted ORs and 95% CIs. These factors included age (per year increase), sex (male *vs*. female), alcohol consumption (ever *vs*. never), smoking (ever *vs*. never), diabetes (yes *vs*. no), and hypertension (yes *vs*. no). These analyses were restricted to any AMD due to insufficient data for AMD subtypes.

#### Regional and provincial prevalence estimation of any AMD in 2020

To estimate regional and provincial prevalence of any AMD, we applied an associated factor-based redistribution model, initially developed by the GHERG. We identified latitude, rural setting, and ever smoking as significantly associated with the prevalence of any AMD in the previous step included them in the model. Province-specific prevalence of rural residence and ever smoking were obtained from the 2020 China national census and a large-scale population-based survey, respectively [12,28]. The latitude of each province was defined using the geographic centroid of its administrative area. Finally, we estimated the number of any AMD cases in each province using the following formula:



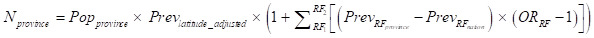



Here, *N_province_* and *Pop_province_* are the number of any AMD cases and population size aged 40–89 years in each province. *Prev_latitude_adjusted_* indicates the estimated national prevalence of any AMD, which is further adjusted for latitude of each province. *RF_1_* and *RF_2_* refer to the two selected associated factors – namely rural setting and ever smoking – from multivariable meta-regression model and meta-analysis of associated factors, respectively. *Prev_RFprovince_* and *Prev_RFnation_* are the prevalence of the two associated factors in each province and Mainland China. *OR_RF_* are the pooled ORs of associated factors.

To ensure consistency, we used an ‘adjustment index’ to ensure that the sum of provincial cases fit within the ‘national envelope’. We calculate the provincial prevalence of any AMD by dividing the case number by the corresponding population for each province, the regional case numbers (for both geographical and economic regions) by aggregating provincial cases, and the regional prevalence by dividing total cases by the respective regional population.

We performed all analyses in *R*, version 4.4.0 (R Core Team, Vienna, Austria). A two-sided *P*-value <0.05 and a 95% CI of OR that did not cross 1.00 was considered statistically significant.

## RESULTS

We retrieved 2294 records from the databases and removed 693 duplicates (**Figure 1**, Panel B). After excluding 1532 ineligible records during title and abstract review, we assessed the full-text of the remaining 69 records. After incorporating 25 articles from the 2017 China AMD Study [9], we included 40 articles in our review, with 24 meeting the criteria for our modelling analysis. Approximately two-thirds of the included articles (n = 25, 62.5%) were published after 2011, and nearly one-third (n = 15, 37.5%) were conducted in rural settings. The quality scores of included studies ranged from 4 to 9, with most studies (n = 28, 70.0%) rated as having high quality (**Figure 1**, Panel C; Table S7–9 and Appendix 4 in the **Online Supplementary Document**).

### National prevalence and case number of any AMD and its subtypes in 2020

**Figure 2:**
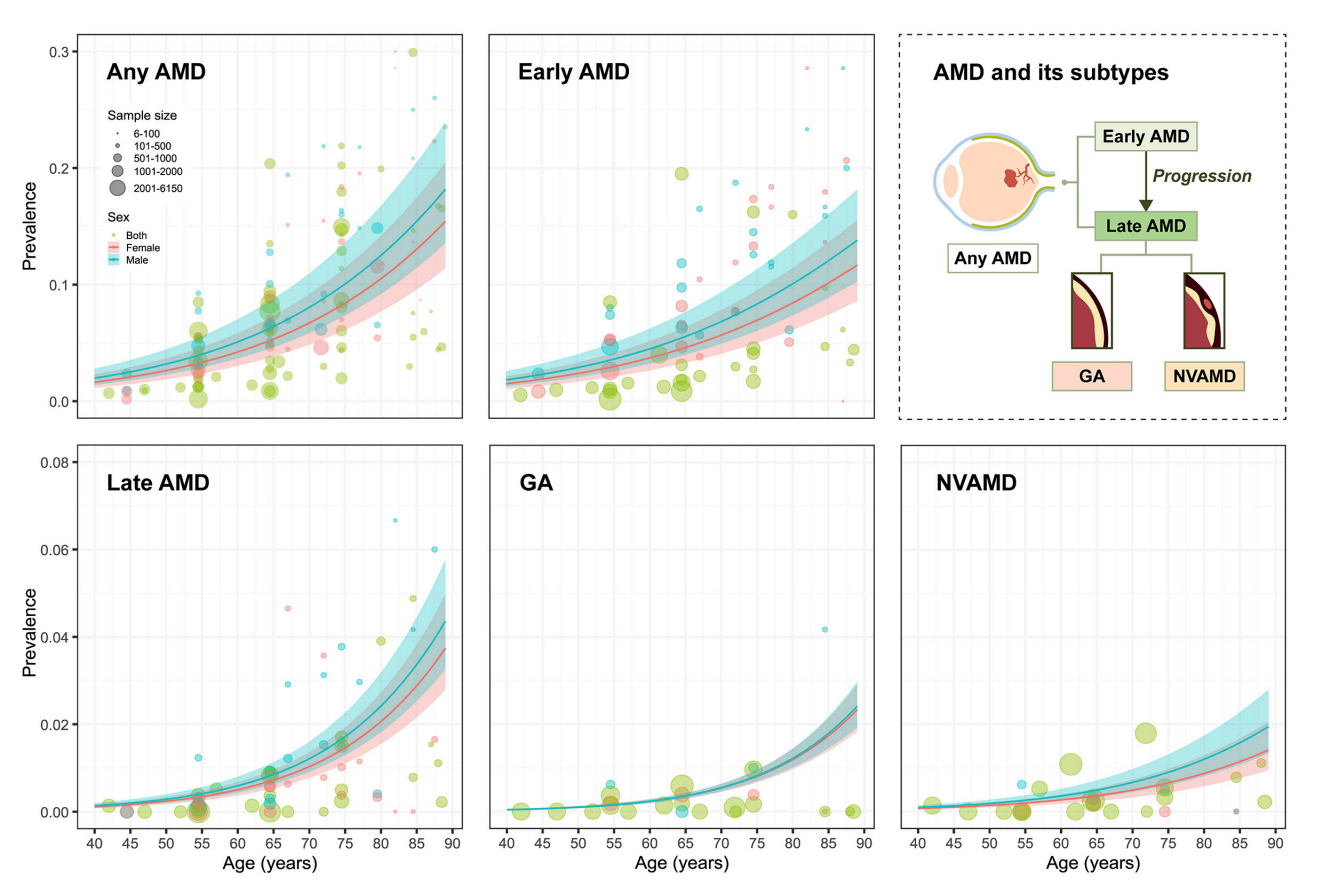
Estimated national age- and sex-specific prevalence of age-related macular degeneration and its subtypes among Chinese people aged 40–89 years in 2020. The size of each bubble is proportional to the sample size of included studies on the prevalence of AMD. AMD – age-related macular degeneration. GA – geographic atrophy, NVAMD – neovascular AMD.

After subtype imputation and age-sex splitting, we conducted a modelling analysis for any AMD and its subtypes, with each analysis using 1978 data points. The overall prevalence of any AMD among individuals aged 40–89 years in Mainland China in 2020 was estimated at 4.70% (95% CI = 3.40, 6.46), corresponding to 32.42 million (95% CI = 23.43, 44.54) affected individuals (**Figure 2**, **Table 1**). A sensitivity analyses that additionally included studies with quality scores <7 or those with an investigation year before 1990 showed a slightly higher, yet largely comparable prevalence of 5.21% (95% CI = 4.12, 6.56) (Table S10 in the **Online Supplementary Document**). The prevalence of any AMD increased progressively with age, from 2.29% (95% CI = 1.63, 3.22) among those aged 40–49 years to 13.28% (95% CI = 9.80, 17.75) in those aged 80–89 years. The overall prevalence was 5.04% (95% CI = 3.65, 6.92) in males and 4.37% (95% CI = 3.15, 6.01) in females, corresponding to 17.31 million (95% CI = 12.53, 23.76) and 15.10 million (95% CI = 10.91, 20.78) cases, respectively (**Table 1**).

**Table 1:** Estimated prevalence and case number of age-related macular degeneration and its subtypes among Chinese people aged 40–89 years in 2020

Age group in years	Any AMD	Early AMD	Late AMD	GA	NVAMD
Both*					
*40–49*	2.29 (1.63, 3.22)	2.11 (1.50, 2.96)	0.18 (0.13, 0.26)	0.07 (0.05, 0.09)	0.12 (0.08, 0.18)
*50–59*	3.56 (2.55, 4.95)	3.19 (2.28, 4.45)	0.36 (0.27, 0.50)	0.15 (0.12, 0.18)	0.22 (0.15, 0.32)
*60–69*	5.78 (4.18, 7.93)	5.00 (3.60, 6.90)	0.77 (0.58, 1.03)	0.35 (0.30, 0.42)	0.42 (0.28, 0.61)
*70–79*	8.77 (6.40, 11.90)	7.29 (5.28, 9.95)	1.48 (1.12, 1.96)	0.75 (0.63, 0.89)	0.73 (0.49, 1.07)
*80–89*	13.28 (9.80, 17.75)	10.44 (7.67, 14.00)	2.84 (2.14, 3.76)	1.60 (1.30, 1.95)	1.24 (0.84, 1.80)
*Overall*	4.70 (3.40, 6.46)	4.06 (2.92, 5.60)	0.64 (0.48, 0.86)	0.30 (0.25, 0.37)	0.34 (0.23, 0.49)
Male*					
*40–49*	2.51 (1.78, 3.52)	2.31 (1.64, 3.23)	0.20 (0.14, 0.28)	0.06 (0.05, 0.09)	0.13 (0.09, 0.20)
*50–59*	3.89 (2.79, 5.40)	3.50 (2.50, 4.86)	0.39 (0.29, 0.54)	0.15 (0.12, 0.18)	0.25 (0.17, 0.36)
*60–69*	6.30 (4.57, 8.64)	5.47 (3.94, 7.53)	0.84 (0.63, 1.11)	0.35 (0.30, 0.41)	0.48 (0.33, 0.70)
*70–79*	9.57 (7.00, 12.95)	7.97 (5.79, 10.84)	1.60 (1.21, 2.11)	0.76 (0.64, 0.88)	0.84 (0.57, 1.23)
*80–89*	14.49 (10.73, 19.29)	11.43 (8.42, 15.24)	3.07 (2.31, 4.05)	1.61 (1.32, 1.95)	1.46 (0.99, 2.10)
*Overall*	5.04 (3.65, 6.92)	4.37 (3.15, 6.02)	0.67 (0.50, 0.90)	0.29 (0.24, 0.35)	0.38 (0.26, 0.55)
Female*					
*40–49*	2.07 (1.47, 2.91)	1.90 (1.35, 2.67)	0.17 (0.12, 0.24)	0.07 (0.05, 0.09)	0.10 (0.07, 0.15)
*50–59*	3.22 (2.31, 4.48)	2.89 (2.06, 4.03)	0.33 (0.24, 0.46)	0.15 (0.12, 0.18)	0.19 (0.13, 0.28)
*60–69*	5.26 (3.80, 7.23)	4.54 (3.26, 6.28)	0.71 (0.53, 0.95)	0.35 (0.29, 0.42)	0.36 (0.24, 0.53)
*70–79*	8.03 (5.85, 10.93)	6.66 (4.81, 9.12)	1.37 (1.04, 1.81)	0.75 (0.62, 0.89)	0.62 (0.41, 0.92)
*80–89*	12.35 (9.08, 16.57)	9.68 (7.08, 13.04)	2.67 (2.00, 3.53)	1.59 (1.28, 1.96)	1.07 (0.72, 1.57)
*Overall*	4.37 (3.15, 6.01)	3.76 (2.70, 5.19)	0.61 (0.45, 0.82)	0.31 (0.26, 0.38)	0.30 (0.20, 0.44)
Both†					
*40–49*	4.75 (3.37, 6.67)	4.37 (3.10, 6.13)	0.38 (0.27, 0.55)	0.13 (0.10, 0.18)	0.25 (0.17, 0.37)
*50–59*	7.92 (5.67, 11.01)	7.11 (5.08, 9.90)	0.81 (0.59, 1.11)	0.33 (0.26, 0.40)	0.49 (0.33, 0.71)
*60–69*	8.51 (6.16, 11.70)	7.37 (5.30, 10.18)	1.14 (0.85, 1.52)	0.52 (0.44, 0.61)	0.62 (0.42, 0.91)
*70–79*	7.09 (5.17, 9.62)	5.89 (4.27, 8.04)	1.20 (0.90, 1.58)	0.61 (0.51, 0.72)	0.59 (0.39, 0.86)
*80–89*	4.15 (3.06, 5.54)	3.26 (2.39, 4.37)	0.89 (0.67, 1.17)	0.50 (0.41, 0.61)	0.39 (0.26, 0.56)
*Overall*	32.42 (23.43, 44.54)	28.00 (20.15, 38.61)	4.42 (3.28, 5.93)	2.09 (1.71, 2.52)	2.33 (1.57, 3.41)
Male†					
*40–49*	2.65 (1.88, 3.72)	2.44 (1.73, 3.42)	0.21 (0.15, 0.30)	0.07 (0.05, 0.09)	0.14 (0.10, 0.21)
*50–59*	4.35 (3.12, 6.05)	3.91 (2.80, 5.44)	0.44 (0.32, 0.61)	0.16 (0.13, 0.20)	0.28 (0.19, 0.40)
*60–69*	4.62 (3.34, 6.33)	4.00 (2.88, 5.51)	0.61 (0.46, 0.81)	0.26 (0.22, 0.30)	0.35 (0.24, 0.51)
*70–79*	3.72 (2.72, 5.04)	3.10 (2.25, 4.22)	0.62 (0.47, 0.82)	0.30 (0.25, 0.34)	0.33 (0.22, 0.48)
*80–89*	1.97 (1.46, 2.62)	1.55 (1.14, 2.07)	0.42 (0.31, 0.55)	0.22 (0.18, 0.26)	0.20 (0.13, 0.29)
*Overall*	17.31 (12.53, 23.76)	15.01 (10.82, 20.67)	2.30 (1.71, 3.09)	1.00 (0.83, 1.20)	1.30 (0.88, 1.89)
Female†					
*40–49*	2.10 (1.49, 2.95)	1.93 (1.37, 2.71)	0.17 (0.12, 0.24)	0.07 (0.05, 0.09)	0.10 (0.07, 0.16)
*50–59*	3.56 (2.55, 4.96)	3.19 (2.28, 4.45)	0.37 (0.27, 0.51)	0.16 (0.13, 0.20)	0.21 (0.14, 0.31)
*60–69*	3.90 (2.82, 5.37)	3.37 (2.42, 4.66)	0.53 (0.40, 0.70)	0.26 (0.22, 0.31)	0.27 (0.18, 0.39)
*70–79*	3.37 (2.45, 4.58)	2.79 (2.02, 3.82)	0.58 (0.43, 0.76)	0.31 (0.26, 0.37)	0.26 (0.17, 0.39)
*80–89*	2.18 (1.60, 2.92)	1.71 (1.25, 2.30)	0.47 (0.35, 0.62)	0.28 (0.23, 0.34)	0.19 (0.13, 0.28)
*Overall*	15.10 (10.91, 20.78)	12.99 (9.33, 17.94)	2.12 (1.57, 2.84)	1.09 (0.88, 1.32)	1.03 (0.69, 1.52)

AMD – age-related macular degeneration, GA – geographic atrophy, NVAMD – neovascular age-related macular degeneration

*Presented as % prevalence (95% confidence interval).

†Presented as number of cases in millions (95% confidence interval).

The prevalence of all AMD subtypes increased with advancing age. The national prevalence of early AMD among individuals aged 40–89 years was 4.06% (95% CI = 2.92, 5.60), with a prevalence of 4.37% (95% CI = 3.15, 6.02) in males and 3.76% (95% CI = 2.70, 5.19) in females. The estimated number of early AMD cases was 28.00 million (95% CI = 20.15, 38.61), with 15.01 million (95% CI = 10.82, 20.67) occurring among males and 12.99 million (95% CI = 9.33, 17.94) among females.

The estimated prevalence of late AMD was 0.64% (95% CI = 0.48, 0.86), affecting 4.42 million (95% CI = 3.28, 5.93) individuals, of whom 2.30 million (95% CI = 1.71, 3.09) were male and 2.12 million (95% CI = 1.57, 2.84) were female. The prevalence of late AMD was 0.67% (95% CI = 0.50, 0.90) in males and 0.61% (95% CI = 0.45, 0.82) in females. Among late AMD subtypes, the overall prevalence of GA and NVAMD was 0.30% (95% CI = 0.25, 0.37) and 0.34% (95% CI = 0.23, 0.49), respectively, amounting to 2.09 million (95% CI = 1.71, 2.52) and 2.33 million (95% CI = 1.57, 3.41) cases, respectively. The prevalence of GA was 0.29% (95% CI = 0.24, 0.35) in males and 0.31% (95% CI = 0.26, 0.38) in females, corresponding to 1.00 million (95% CI = 0.83, 1.20) and 1.09 million (95% CI = 0.88, 1.32) cases, respectively. For NVAMD, the prevalence was 0.38% (95% CI = 0.26, 0.55) in males and 0.30% (95% CI = 0.20, 0.44) in females, accounting for 1.30 million (95% CI = 0.88, 1.89) and 1.03 million (95% CI = 0.69, 1.52) cases, respectively.

### Effect sizes of factors associated with any AMD

Multivariable meta-regression adjusted for age and sex revealed that latitude was significantly associated with any AMD, with a negative coefficient of −0.0560 (95% CI = −0.1013, −0.0106; *P* = 0.0155). Rural settings, compared with urban ones, were also associated with lower prevalence of any AMD (*β* = −0.1281; 95% CI = −0.2516, −0.0047; *P* = 0.0420) (Table S5 in the **Online Supplementary Document**). Among the individual-level factors included in the random-effects meta-analyses (Table S6 in the **Online Supplementary Document**), older age was associated with a significantly higher odds of any AMD (OR = 1.04; 95% CI = 1.00, 1.08 per year increase), as was ever smoking (OR = 2.42; 95% CI = 1.04, 5.64).

### Regional prevalence and case number of any AMD in 2020

**Figure 3:**
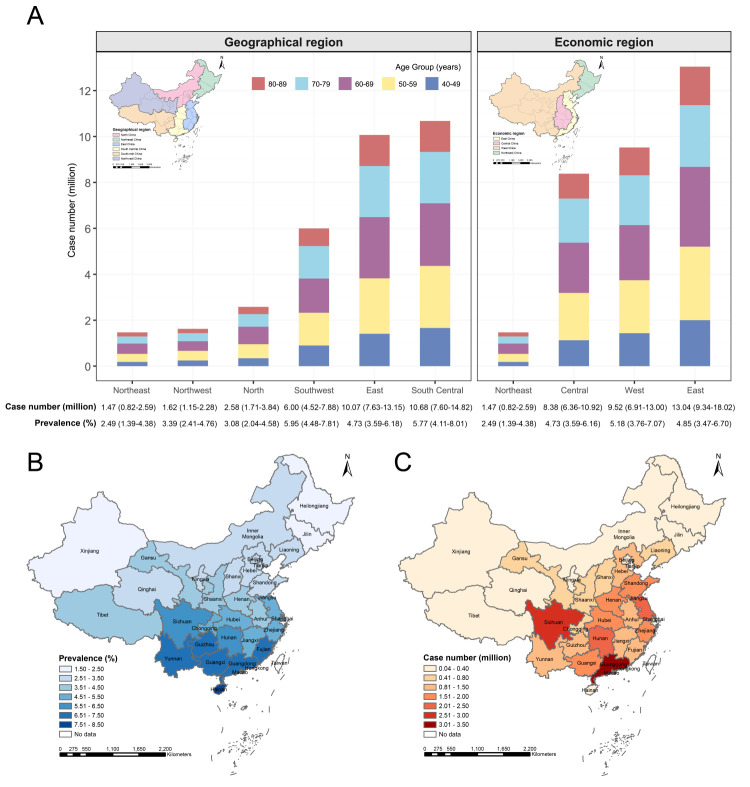
Estimated regional and provincial prevalence and case number of any age-related macular degeneration among Chinese people aged 40–89 years in 2020. Data are presented with prevalence (%) or case number (million) and 95% confidence interval. **Panel A.** Estimated regional prevalence and case number. **Panel B.** Estimated provincial prevalence. **Panel C.** Estimated provincial case number.

Across the six geographical regions of Mainland China (**Figure 3**, Panel A; Table S11 in the **Online Supplementary Document**), we observed the highest prevalence of any AMD in Southwest China (5.95%; 95% CI = 4.48, 7.81), and the lowest in Northeast China (2.49%; 95% CI = 1.39, 4.38). South Central China and Northeast China bore the most and fewest cases of any AMD, at 10.68 million (95% CI = 7.60, 14.82) and 1.47 million (95% CI = 0.82, 2.59), respectively. Among the four economic regions (**Figure 3**, Panel A; Table S12 in the **Online Supplementary Document**), West China and Northeast China exhibited the highest (5.18%; 95% CI = 3.76, 7.07) and lowest (2.49%; 95% CI = 1.39, 4.38) prevalence, while East China and Northeast China had the most (13.04 million; 95% CI = 9.34, 18.02) and fewest cases (1.47 million; 95% CI = 0.82, 2.59), respectively.

### Provincial prevalence and case number of any AMD in 2020

We noted the highest prevalence of any AMD in Hainan (7.64%; 95% CI = 4.61, 12.22), and the lowest in Xinjiang (1.96%; 95% CI = 1.07, 3.51) (**Figure 3**, Panel B; Table S13 in the **Online Supplementary Document**). Fujian (8.38%; 95% CI = 6.08, 11.37) and Heilongjiang (1.83%; 95% CI = 0.92, 3.54) showed the highest and lowest prevalence in males, respectively (Figure S5 in the **Online Supplementary Document**) Conversely, the highest and lowest prevalence among females was 7.76% (95% CI = 4.68, 12.42) in Hainan and 2.07% (95% CI = 1.13, 3.71) in Xinjiang. Moreover, Guangdong accounted for the largest share of individuals affected by any AMD (3.50 million; 95% CI = 2.34, 5.13), with 1.95 million (95% CI = 1.30, 2.86) cases in males and 1.55 million (95% CI = 1.04, 2.27) in females (**Figure 3**, Panel C). Tibet bore the fewest cases (0.04 million; 95% CI = 0.03, 0.06) with equivalent estimates of 0.02 million (95% CI = 0.02, 0.03) for males and females, each (Figure S5 in the **Online Supplementary Document**).

## DISCUSSION

This systematic review and modelling analysis presents the most comprehensive and up-to-date estimates of the prevalence and number of individuals with AMD in Mainland China at national, regional, and provincial levels. The prevalence of any AMD among individuals aged 40–89 years in China in 2020 was 4.70%, corresponding to approximately 32.42 million cases. Of these, 28.00 million (86.4%) had early AMD, while 4.42 million (13.6%) had late AMD, including 2.09 million with GA and 2.33 million with NVAMD. The burden of AMD increased substantially with age and was unevenly distributed by sex and geography, underscoring the growing significance of AMD as a public health priority in the context of China’s rapidly ageing population.

The age-related patterns observed here were consistent with the well-established biological association between AMD and ageing, reflecting the degenerative nature of the disease and age-related retinal changes [11]. This pattern was consistent across both sexes and highlighted the importance of age-targeted screening and early detection strategies. However, we did observe some sex-specific differences in AMD prevalence, whereby the prevalence of any AMD and all subtypes except GA was higher in males than females. This pattern is consistent with several prior studies and potentially attributable to higher exposure to risk factors such as smoking among males [29–31]. Furthermore, females accounted for a higher number of cases among individuals aged 80–89 years, likely reflecting longer female life expectancy and a larger at-risk population at older ages [32]. These findings emphasise the need to consider both age and sex in AMD healthcare planning, particularly in light of China’s demographic transition.

Although early AMD accounted for the majority of cases, late AMD (namely GA and NVAMD) has disproportionate implications for irreversible vision loss and healthcare costs. The estimated national prevalence of GA and NVAMD was 0.30% and 0.34%, respectively. Currently, no approved therapy exists for GA [2], and although NVAMD is treatable with intravitreal anti-vascular endothelial growth factor therapy, treatment remains costly, requires frequent injections, and is thus less accessible in low-resource settings [3]. With population ageing, the absolute number of individuals affected by late AMD will increase substantially, necessitating investments in access to treatment, long-term care, and vision rehabilitation services. Furthermore, emerging evidence suggests pathophysiological overlap between GA and NVAMD, with potential for progression between subtypes over time [33,34]. The similarity in prevalence between GA and NVAMD in this study reinforces the need for greater clinical and research attention to both forms of late AMD.

Geographic variation in prevalence and case numbers of any AMD was substantial across China. At the regional level, Southwest China had the highest prevalence, while Northeast China had the lowest. Among economic regions, West China recorded the highest prevalence, whereas East China, home to a large and ageing population, accounted for the greatest number of cases. These findings underscore the importance of differentiating between relative and absolute burden when prioritising resource allocation. At the provincial level, Hainan had the highest prevalence of any AMD overall and among females, while Fujian had the highest prevalence in males, potentially reflecting behavioural and demographic differences, including high smoking rates in Fujian [12]. In contrast, populous provinces such as Guangdong carried a disproportionately large share of the national AMD burden due to the combined effects of population size and ageing. Sparsely populated provinces like Tibet, despite lower total case numbers, should not be overlooked given the challenges posed by limited healthcare access [35]. Recognising these subnational disparities is critical for designing equitable, efficient, and targeted eye health interventions.

Several contextual and behavioural factors were significantly associated with AMD prevalence. Latitude was inversely associated with any AMD prevalence, indicating higher disease burden in lower-latitude (southern) provinces. This may reflect greater cumulative UV radiation exposure, but could also be related to environmental and dietary factors specific to southern China [36–38]. Additionally, individuals residing in rural settings had a slightly lower prevalence of AMD compared those living in urban settings. This may be explained by differences in lifestyle, occupational exposure, or dietary patterns. Rural residents are more likely to engage in physical labour, consume less processed food, and have lower screen time, all of which have been associated with reduced risk of AMD [20,39–41]. Consistent with global findings, we found ever smoking to be a strong individual-level associated factor for AMD, likely due to oxidative stress, vascular dysfunction, and inflammation induced by tobacco exposure [42]. Taken together, these findings underscore the need for more granular and behaviourally informed exposure assessments in future research to improve risk modelling and subnational burden estimation.

This study has several strengths. It is the first to provide a robust, standardised synthesis of AMD prevalence and case numbers across all 31 provincial-level units in Mainland China. It included only population-based studies with clearly defined diagnostic criteria, ensuring the quality and comparability of input data. The modelling approach maximised the use of available evidence through subtype imputation and age-specific and sex-specific splitting. This is particularly important given that large-scale epidemiological studies in ophthalmology are relatively uncommon, largely due to the high costs and technical demands of ocular assessments. In the absence of nationally representative eye health surveys covering all provinces, this modelling strategy offers a practical and timely alternative to inform health system planning. Beyond the specific context of China, our study offers a methodological framework for improving global equity in eye health. Many low- and middle-income countries share the dual challenge of rapid population ageing and limited empirical data. In such settings, model-based estimation provides a cost-effective means to assess disease burden and guide policy for the prevention of vision loss. This is critical for equitable resource allocation, directing eye health interventions to underserved, high-burden populations instead of solely to regions with established research infrastructure.

Nevertheless, several limitations should be acknowledged. First, despite the use of rigorous inclusion criteria, heterogeneity in AMD grading systems and case definitions across studies may have introduced variability. While we applied standardisation and imputation methods to mitigate this bias, the CIs may not fully capture the additional uncertainty specifically introduced by these procedures. Second, the restriction of high-quality studies for modelling, while ensuring internal validity, inevitably exacerbated geographic data gaps. Data were unavailable for some provinces, particularly in Western and Northeast China, necessitating extrapolation from modelled covariates rather than direct estimation. Although sensitivity analyses suggested this did not skew overall national estimates, it may have reduced the precision of estimates at the subnational level. Third, the model did not incorporate several potentially important AMD risk factors, such as diet, physical activity, and occupational exposures, due to the limited availability of relevant data. Finally, while we were able to estimate regional and provincial prevalence for any AMD, the scarcity of data on associated factors for AMD subtypes limited our ability to assess their geographic distribution. This underscores the need for more detailed, subtype-specific epidemiological research to strengthen future modelling efforts and guide targeted public health interventions.

## CONCLUSIONS

Our study shows a substantial public health burden of AMD in Mainland China in 2020, affecting over 32 million adults aged 40–89 years. We noted significant geographic and demographic heterogeneity, with important implications for health system planning, prioritisation, and service delivery. The strong associations with age, smoking, and environmental context highlight a need for regionally tailored, integrated approaches to AMD prevention, detection, and management. As China continues to undergo rapid population ageing, incorporating AMD into national eye health and healthy ageing strategies will be critical to reducing vision loss and improving quality of life for older adults.

## Additional material


Online Supplementary Document
