# Supplementary material for: National, regional, and provincial prevalence of age-related macular degeneration in China in 2020: an updated systematic review and modelling study
Source: J Glob Health. 2026 Jan 23;16:04062. doi: 10.7189/jogh.16.04062 (PMC12829514; doi:10.7189/jogh.16.04062)
Supplement: Online Supplementary Document [file jogh-16-04062-s001.pdf]

Supplement to: Wu J, Shan S, Zhou J, Li Y, Ke Q, Zhu L, Rudan I, Song P; Global Health Epidemiology Research Group. National, regional, and provincial prevalence of age-related macular degeneration in China in 2020: an updated systematic review and modelling study. J Glob Health. 2026;16:04062.

Appendix 1. Search strategy to identify articles reporting the prevalence of age-related macular degeneration in China 2
Appendix 2. eMethods: The detailed description of age-sex splitting of the prevalence of age-related macular degeneration (AMD) 5
Appendix 3. Supplementary tables and figures 7
- **Table S1. The geographical and economic regions in the Mainland of China** 7
- **Table S2. The time-lag between investigation and publication in the included studies** 8
- **Table S3. Joanna Briggs Institute (JBI) critical appraisal checklist for studies reporting prevalence data** 10
- **Table S4. Basic characteristics of the studies used for subtype imputation (n=13)** 11
- **Table S5. Age- and sex- adjusted multi-level mixed-effects meta-regression models for variables of interest on the prevalence of any AMD** 12
- **Table S6. Meta-analysis of associated factors of any AMD** 13
- **Table S7. Detailed characteristics of included articles (n=40)** 15
- **Table S8. Quality scores for assessing the risk of bias in the included studies (n=40)** 17
- **Table S9. Summary of the main characteristics of the included studies (n=40)** 19
- **Table S10. Sensitivity analysis of estimated prevalence (%) and case number (million) of any AMD in the Mainland of China in 2020 (additionally including studies with quality scores <7 or investigation year before 1990)** 20
- **Table S11. Estimated prevalence (%) and case number (million) of any AMD by geographical regions in the Mainland of China in 2020** 21
- **Table S12. Estimated prevalence (%) and case number (million) of any AMD by economic regions in the Mainland of China in 2020** 24
- **Table S13. Estimated provincial prevalence (%) and case number (million) of any AMD in the Mainland of China in 2020** 26
- **Figure S1. Meta-analysis of the early AMD to any AMD ratio for subtype imputation** 28
- **Figure S2. Meta-analysis of the late AMD to any AMD ratio for subtype imputation** 29
- **Figure S3. Meta-analysis of the GA to late AMD ratio for subtype imputation** 30
- **Figure S4. Meta-analysis of the NVAMD to late AMD ratio for subtype imputation** 31
- **Figure S5. Estimated provincial prevalence (%) and case number (million) of any AMD among individuals aged 40-89 years in the Mainland of China in 2020** 32

Appendix 4. Full list of the included articles (n=40) 33
Appendix 5. PRISMA 2020 reporting checklist 37

Appendix 1. Search strategy to identify articles reporting the prevalence of age-related macular degeneration in China

Database	Access date	Subject category	Sub-database	Search terms	Publication date	Search method
**CNKI**	30/7/2024	Medicine & Public Health	Journal, Featured journal, Doctoral dissertation, Master dissertation, Domestic conferences, International conferences	(SU %='年龄相关黄斑变性' + '年龄相关性黄斑变性' + '老年黄斑变性' + '老年性黄斑变性' + '年龄相关黄斑病变' + '年龄相关性黄斑病变' + '老年黄斑病变' + '老年性黄斑病变') AND (SU %='发病率' + '发生率' + '患病率' + '罹患率' + '现患率' + '死亡率' + '病死率' + '流行' + '负担' + '现况调查' + '现况研究')	27/06/2016-30/07/2024	Comprehensive search: subject, title, keywords and abstract
**Wanfang**	30/7/2024	Not applicable	Journal articles, Dissertations, Conference articles	(主题:(年龄相关黄斑变性) or 主题:(年龄相关性黄斑变性) or 主题:(老年黄斑变性) or 主题:(老年性黄斑变性) or 主题:(年龄相关黄斑病变) or 主题:(年龄相关性黄斑病变) or 主题:(老年黄斑病变) or 主题:(老年性黄斑病变)) AND (主题:(发病率) or 主题:(发生率) or 主题:(患病率) or 主题:(罹患率) or 主题:(现患率) or 主题:(死亡率) or 主题:(病死率) or 主题:(流行) or 主题:(负担)or 主题:(现况调查) or 主题:(现况研究))	2016-2024	Comprehensive search: subject (including title, keywords and abstract)
**VIP**	30/7/2024	Medicine & Public Health	All journals	((M=(年龄相关黄斑变性 OR 年龄相关性黄斑变性 OR 老年黄斑变性 OR 老年性黄斑变性 OR 年龄相关黄斑病变 OR 年龄相关性黄斑病变 OR 老年黄斑病变 OR 老年性黄斑病变)) OR (R=(年龄相关黄斑变性 OR 年龄相关性黄斑变性 OR 老年黄斑变性 OR 老年性黄斑变性 OR 年龄相关黄斑病变 OR 年龄相关性黄斑病变 OR 老年黄斑病变 OR 老年性黄斑病变))) AND ((M=(发病率 OR 发生率 OR 患病率 OR 罹患率 OR 现患率 OR 死亡率 OR 病死率 OR 流行 OR 负担 OR 现况调查 OR 现况研究)) OR (R=(发病率 OR 发生率 OR 患病率 OR 罹患率	2016-2024	Comprehensive search: subject, title, keywords and abstract

Database	Access date	Subject category	Sub-database	Search terms	Publication date	Search method
				OR 现患率 OR 死亡率 OR 病死率 OR 流行 OR 负担 OR 现况调查 OR 现况研究)))		
**PubMed**	30/7/2024	Not applicable	Not applicable	((age-related macular degeneration OR age related macular degeneration OR age-related maculopathy OR age related maculopathy) AND (China OR Chinese OR Hongkong OR Macao OR Taiwan) AND (inciden* OR prevalen* OR morbidity OR mortality OR epidemiology)) AND ("2016/06/27"[Date - Publication] : "2024/07/30"[Date - Publication])	27/06/2016-30/07/2024	Comprehensive search: all fields
**Embase**	30/7/2024	Not applicable	Not applicable	#1 'age-related macular degeneration':ab,ti OR 'retina macula age related degeneration'/exp OR 'age related macular degeneration'/exp OR 'retina macular degeneration'/exp OR 'retina maculopathy'/exp OR 'age-related maculopathy':ab,ti<br>#2 'China':ab,ti OR 'China'/exp OR 'Chinese':ab,ti OR 'Chinese'/exp OR 'Hong Kong':ab,ti OR 'Hong Kong'/exp OR 'Macao':ab,ti OR 'Macao'/exp OR 'Taiwan':ab,ti OR 'Taiwan'/exp<br>#3 'inciden*':ab,ti OR 'incidence'/exp OR 'prevalen*':ab,ti OR 'prevalence'/exp OR 'morbidity*':ab,ti OR 'morbidity'/exp OR 'mortality':ab,ti OR 'mortality'/exp OR 'epidemiolog*':ab,ti OR 'epidemiology'/exp<br>#4 #1 AND #2 AND #3<br>#5 #4 AND [embase]/lim NOT ([embase]/lim AND [medline]/lim)<br>#6 #5 AND [2016-2024]/py	2016-2024	Comprehensive search: all fields
**MEDLINE**	30/7/2024	Not applicable	Not applicable	#1 age-related macular degeneration.mp. or exp Macular Degeneration/ or age-related maculopathy.mp.	2016-2024	Comprehensive search: all fields

Database	Access date	Subject category	Sub-database	Search terms		Publication date	Search method
				#2	China.mp. or exp China/ or Chinese.mp. or exp Chinese/ or Hong Kong.mp. or exp Hong Kong/ or Macao.mp. or exp Macau/ or Taiwan.mp. or exp Taiwan/		
				#3	exp incidence/ or inciden*.mp. or exp prevalence/ or prevalent*.mp. or morbidity.mp. or exp morbidity/ or mortality.mp. or exp mortality/ or epidemiolog*.mp. or exp epidemiology/		
				#4	1 and 2 and 3		
				#5	limit 4 to yr="2016 -Current"		

Number of records returned: 2294.

Appendix 2. eMethods: The detailed description of age-sex splitting of the prevalence of age-related macular degeneration (AMD)

This section is a supplement to the Methods part in the main text.

1 Age- and sex- prevalence patterns of any AMD and its subtypes

An age-sex splitting method was conducted to split aggregated prevalence data into age- and sex-specific estimates. To provide the basis for age-sex splitting, multi-level mixed-effects meta-regression models were utilized to establish the association of age and sex with the prevalence and to generate the corresponding "prevalence patterns". The effect of datapoints clustering from the same study or the same province was controlled by adding study and province identification into the regression model as the random effect. Given that,

$$prevalence = p = \frac{case\ number\ of\ any\ AMD\ or\ its\ subtypes}{number\ of\ participants}$$

Then the prevalence estimates were transformed by the logit link, while a nominal value of 0.0005 was assigned to zero-case studies to facilitate the transformation.

$$logit(p) = \ln\left(\frac{p}{1-p}\right) = \ln(odds) = \alpha + \beta_1 * average\ age + \beta_2 * female\ proportion + u_i$$

Therefore,

$$odds = \frac{p}{1-p} = e^{(\alpha+\beta_1 * average\ age + \beta_2 * female\ proportion + u_i)}$$

And,

$$prevalence = p = \frac{e^{(\alpha+\beta_1 * average\ age + \beta_2 * female\ proportion + u_i)}}{1 + e^{(\alpha+\beta_1 * average\ age + \beta_2 * female\ proportion + u_i)}}$$

Where p is the prevalence, α is the intercept term, β is the coefficient, and u_i reflects the random effects at the study and province level, controlling for clustering of multiple data points from the same study or province.

Based on the above models, the age- and sex- "prevalence patterns" were generated.

2 Age-sex splitting for prevalence of any AMD and its subtypes

Based on the "prevalence patterns", we employed an age-sex splitting approach to convert aggregated data to standard age and sex groups. First, a sex splitting was performed on datapoints specified as "both" sex into male- and female-specific datapoints. The following equation was employed:

$$C_{A,s} = (\sum_{a \in A} R_{a,s} N_{a,s}) \cdot P_{A,s}/N_{A,s} \cdot \frac{C_{A,S}}{\sum_{s \in S}((\sum_{a \in A} R_{a,s} N_{a,s}) \cdot P_{A,s}/N_{A,s})}$$

In this equation, s is the specific sex (male or female), S is the set of sexes that the data is aggregated across, a is a one-year age group, A is the set of ages that the data is aggregated across, $C_{A,S}$ is the reported total case number to be split, $R_{a,s}$ is the prevalence in age group a and sex s

from “prevalence patterns”, $N_{a,s}$ is the population in age group a and sex s based on the 2020 population census of China, $P_{A,s}$ is the proportion of sex s in study sample, $N_{A,s}$ is the population in age group A and sex s based on the 2020 population census of China, and $C_{A,s}$ is the case number of any AMD or its subtypes in sex s after splitting.

Subsequently, datapoints with inconsistent age groups were split into uniform one-year age groups. Given the lack of precise age-specific distribution data in the included studies, we employed an exponential adjustment method with numerical optimization to simulate the age distributions align with both the national age distribution and the sample’s average age.

We introduced an adjustment parameter adj to modify the national age distribution using an exponential function:

$$Adjusted\ P_a = \frac{P_a \cdot e^{adj(a-\mu)}}{\sum_{a \in A} P_a \cdot e^{adj(a-\mu)}}$$

where a is a one-year age group, A is the set of ages that the data is aggregated across, adj is the adjustment parameter, μ is the average age of reported sample, P_a is the proportion of age a in the 2020 population census of China, and $Adjusted\ P_a$ is the proportion of age a after adjustment. This adjustment increases the proportion of ages above μ if adj >0 and increases the proportion of ages below μ if adj <0.

The optimal value of adj were estimated using the Brent optimization method to minimize the squared difference between the average age of the adjusted distribution and the reported average age:

$$\min_{adj} \left(\sum_{a \in A} Adjusted\ P_a \cdot a - \mu \right)^2$$

The optimization was constrained to $adj \in [-10, 10]$ to ensure numerical stability.

After obtaining the optimal adj, we generated the adjusted age distribution $Adjusted\ P_a$. We then sampled ages from this distribution to create a simulated population that aligns with both the national age distribution and the target mean age.

An age splitting was then performed based on the adjusted age distribution:

$$C_a = R_a N_a \frac{C_A}{\sum_{a \in A} R_a N_a}$$

In this equation, a is a one-year age group, A is the set of ages that the data is aggregated across, C_A is the reported total case number in ages A to be split, R_a is the prevalence in age group a from “prevalence patterns”, N_a is the population in age group a based on the simulated population, and C_a is the split case number of any AMD or its subtypes in age group a.

After performing age-sex splitting, the case numbers for any AMD or its subtypes from various studies were divided into single-year age groups and separated by sex (male and female).

Appendix 3. Supplementary tables and figures

Table S1. The geographical and economic regions in the Mainland of China

**Region**	**Included provinces**
Geographical regions	
North China	Beijing Municipality, Hebei province, Inner Mongolia Autonomous Region, Shanxi province, Tianjin Municipality
Northeast China	Heilongjiang province, Jilin province, Liaoning province
East China	Anhui province, Fujian province, Jiangsu province, Jiangxi province, Shandong province, Shanghai Municipality, Zhejiang province
South Central China	Guangdong province, Guangxi Zhuang Autonomous Region, Hainan province, Henan province, Hubei province, Hunan province
Southwest China	Chongqing Municipality, Guizhou province, Sichuan province, Tibet Autonomous Region, Yunnan province
Northwest China	Gansu province, Ningxia Hui Autonomous Region, Qinghai province, Shaanxi province, Xinjiang Uyghur Autonomous Region
Economic regions	
East China	Beijing Municipality, Hebei province, Tianjin Municipality, Shanghai Municipality, Jiangsu province, Zhejiang province, Fujian province, Shandong province, Guangdong province, Hainan province
Central China	Shanxi province, Anhui province, Jiangxi province, Henan province, Hubei province, Hunan province
West China	Inner Mongolia Autonomous Region, Guangxi Zhuang Autonomous Region, Chongqing Municipality, Sichuan province, Guizhou province, Tibet Autonomous Region, Yunnan province, Shaanxi province, Gansu province, Qinghai province, Ningxia Hui Autonomous Region, Xinjiang Uyghur Autonomous Region
Northeast China	Heilongjiang province, Jilin province, Liaoning province

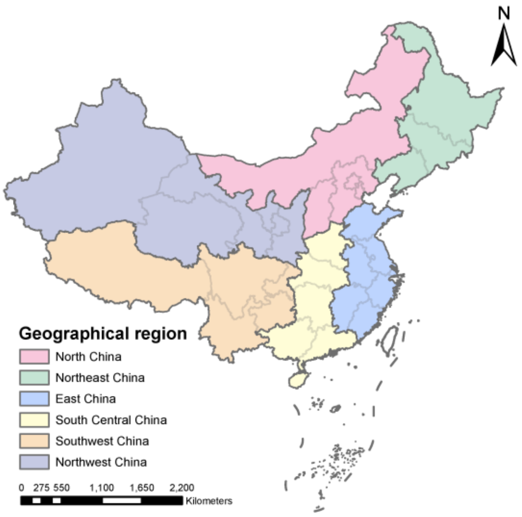

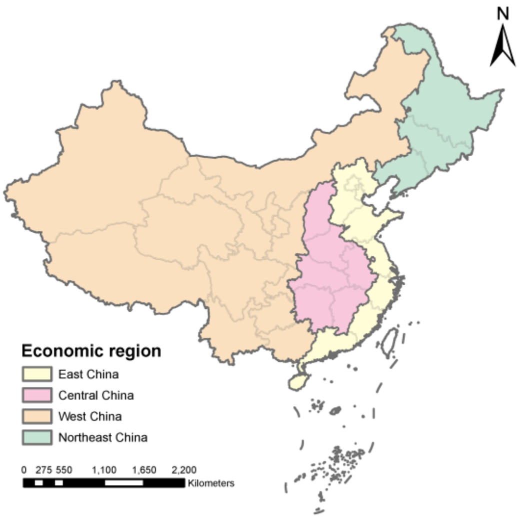

Table S2. The time-lag between investigation and publication in the included studies

Study ID	Author	Publication year	investigation year	Time-lag (years)
AMD-01	Huang P, et al.	1992	1987	5
AMD-02	Tan JQ, et al.	1992	NA	NA
AMD-03	Wu ZQ, et al.	1992	1989	3
AMD-04	Chen YH, et al.	1993	1991	2
AMD-05	Yu Q, et al.	1994	1990	4
AMD-06	Xuan MZ, et al.	1994	1989	5
AMD-07	He MG, et al.	1998	1997	1
AMD-08	Zou HD, et al.	2005	2003	2
AMD-09	Tian MN, et al.	2005	NA	NA
AMD-10	Bai ZL, et al.	2005	2003	2
AMD-11	Shi K, et al.	2009	2006	3
AMD-12	Li HL, et al.	2009	2009	0
AMD-13	Zhao X, et al.	2011	2006	5
AMD-14	Huang XB, et al.	2012	2008	4
AMD-15	Yang Z.	2014	2013	1
AMD-16	Cao J.	2014	2010	4
AMD-17	Xiang W, et al.	2015	2014	1
AMD-18	Wang Y, et al.	2015	2015	0
AMD-19	Li WL, et al.	2015	2013	2
AMD-20	Li Y, et al.	2006	2001	5
AMD-21	Chen SJ, et al.	2008	1999	9
AMD-22	Huang TL, et al.	2010	NA	NA
AMD-23	Yang K, et al.	2011	2006	5
AMD-24	Ye H, et al.	2014	2013	1
AMD-25	Huang EJ, et al.	2014	2011	3
AMD-26	Wang H, et al.	2018	NA	NA
AMD-27	Liu JW, et al.	2019	2017	2
AMD-28	Bai QX, et al.	2023	2018	5
AMD-29	Zhang YL, et al.	2020	2019	1
AMD-30	Mai JY, et al.	2017	2013	4
AMD-31	Guan Y, et al.	2018	2011	7
AMD-32	Xu HM, et al.	2018	2015	3
AMD-33	Zhang XY, et al.	2022	2019	3
AMD-34	Guan RJ, et al.	2020	NA	NA
AMD-35	Jin GM, et al.	2017	2014	3
AMD-36	Zhang KY, et al.	2017	2015	2
AMD-37	Wang HJ, et al.	2017	2016	1
AMD-38	Xi C, et al.	2017	2010	7
AMD-39	Lin YH, et al.	2022	2018	4
AMD-40	Li Y, et al.	2023	2020	3

Notes: NA, not available. The average time-lag between investigation and publication was 3.2 years

based on 35 studies with available data.

Table S3. Joanna Briggs Institute (JBI) critical appraisal checklist for studies reporting prevalence data

Question
1. Was the sample frame appropriate to address the target population?
2. Were study participants sampled in an appropriate way?
3. Was the sample size adequate?
4. Were the study subjects and the setting described in detail?
5. Was the data analysis conducted with sufficient coverage of the identified sample?
6. Were valid methods used for the identification of the condition?
7. Was the condition measured in a standard, reliable way for all participants?
8. Was there appropriate statistical analysis?
9. Was the response rate adequate, and if not, was the low response rate managed appropriately?

Table S4. Basic characteristics of the studies used for subtype imputation (n=13)

Study ID	Author (s)	Publication year	Province	Setting	Study year	Sample size	Female proportion	Any AMD	Early AMD	Late AMD	GA	NVAMD
AMD-13	Zhao X, et al.	2011	Beijing	Urban	2006	2348	0.5281	77	70	7	3	4
AMD-15	Yang Z.	2014	Sichuan	Urban	2013	2097	0.6433	207	194	13	9	4
AMD-17	Xiang W, et al.	2015	Ningxia	Rural	2014	4812	0.4994	54	46	8	-	-
AMD-18	Wang Y, et al.	2015	Jiangsu	Rural	2015	2985	0.6549	97	71	26	17	9
AMD-19	Li WL, et al.	2015	Ningxia	Rural	2013	679	0.4654	25	17	8	-	-
AMD-20	Li Y, et al.	2006	Beijing	Mixed	2001	4376	0.5643	70	63	7	1	6
AMD-21	Chen SJ, et al.	2008	Taiwan	Mixed	1999	1058	0.3771	117	97	20	1	19
AMD-23	Yang K, et al.	2011	Hebei	Rural	2006	6581	0.5347	204	200	4	-	-
AMD-24	Ye H, et al.	2014	Shanghai	Urban	2013	2005	0.5626	229	206	23	-	-
AMD-25	Huang EJ, et al.	2014	Taiwan	Mixed	2011	673	0.4577	150	101	49	-	-
AMD-27	Liu JW, et al.	2019	Xinjiang	Rural	2017	1202	0.5308	73	48	25	12	13
AMD-35	Jin GM, et al.	2017	Guangdong	Rural	2014	4881	0.5087	2924	2882	42	-	-
AMD-36	Zhang KY, et al.	2017	Hainan	Mixed	2015	2232	0.6564	357	331	26	-	-

Notes: AMD, age-related macular degeneration; GA, geographic atrophy; NVAMD, neovascular AMD.

Table S5. Age- and sex- adjusted multi-level mixed-effects meta-regression models for variables of interest on the prevalence of any AMD

Variable	Number of studies	*β* (95% CI)	*P*-value
Publication year	24	-0.0105 (-0.0470, 0.0260)	0.5742
Investigation year	24	-0.0136 (-0.0462, 0.0191)	0.4153
Latitude	24	-0.0560 (-0.1013, -0.0106)	0.0155
Longitude	24	-0.0047 (-0.0394, 0.0300)	0.7917
Altitude	24	-0.0004 (-0.0012, 0.0004)	0.3039
Insolation	24	-0.1915 (-0.8208, 0.4379)	0.5510
Setting			
Urban	9	Reference	
Rural	11	-0.1281 (-0.2516, -0.0047)	0.0420

Notes: AMD, age-related macular degeneration; CI, confidence interval. Each row represents the result from a separate meta-regression model, which included age and sex as covariates along with the specific variable listed in that row.

Table S6. Meta-analysis of associated factors of any AMD

Study ID	Author (s)	Publication year	Sample size	Pooled OR (95% CI)	Forest Plot	Funnel Plot	Sensitivity analysis
Age (per year increase)							
AMD-16	Jia Cao.	2014	6150	1.04 (1.00, 1.08)	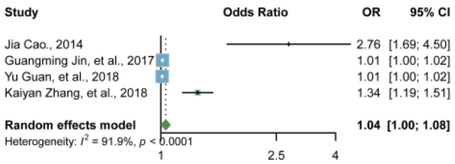	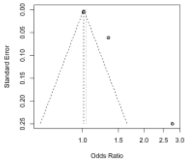	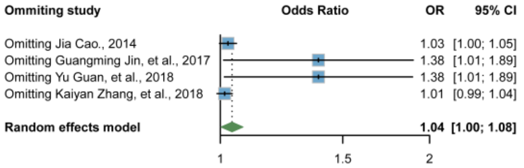
AMD-35	Guangming Jin, et al.	2017	4881				
AMD-31	Yu Guan, et al.	2018	5947				
AMD-36	Kaiyan Zhang, et al.	2018	2232				
Sex (male vs female)							
AMD-10	Zhilan Bai, et al.	2005	2835	1.08 (0.79, 1.47)	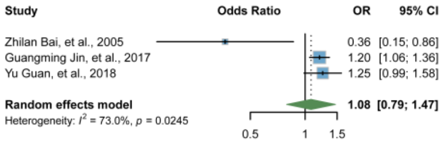	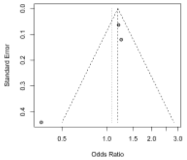	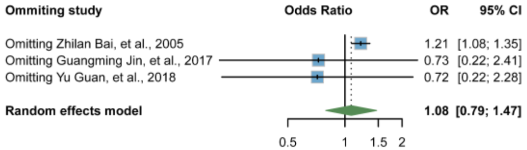
AMD-35	Guangming Jin, et al.	2017	4881				
AMD-31	Yu Guan, et al.	2018	5947				
Alcohol consumption (ever vs never)							
AMD-10	Zhilan Bai, et al.	2005	2835	1.20 (0.58, 2.48)	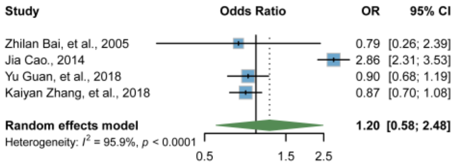	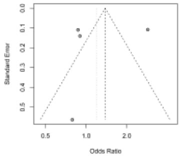	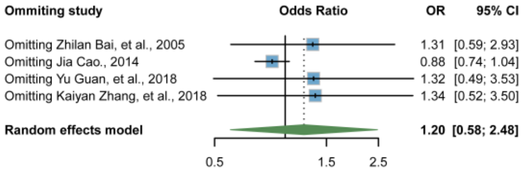
AMD-16	Jia Cao.	2014	6150				
AMD-31	Yu Guan, et al.	2018	5947				
AMD-36	Kaiyan Zhang, et al.	2018	2232				
Smoking (ever vs never)							
AMD-10	Zhilan Bai, et al.	2005	2835	2.42 (1.04, 5.64)	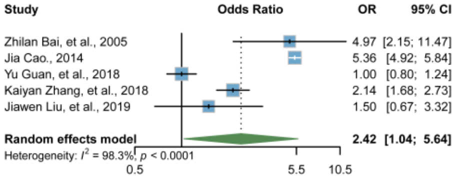	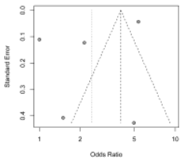	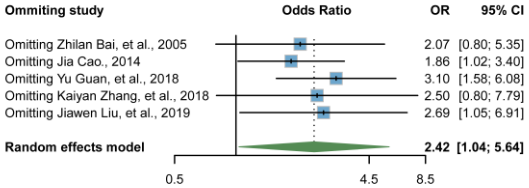
AMD-16	Jia Cao.	2014	6150				
AMD-31	Yu Guan, et al.	2018	5947				
AMD-36	Kaiyan Zhang, et al.	2018	2232				

AMD-27	Jiawen Liu, et al.	2019	1202	
Diabetes (yes vs no)				
AMD-16	Jia Cao.	2014	6150	
AMD-35	Guangming Jin, et al.	2017	4881	0.92 (0.74, 1.14)
AMD-31	Yu Guan, et al.	2018	5947	
AMD-27	Jiawen Liu, et al.	2019	1202	

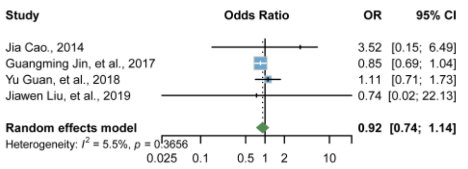

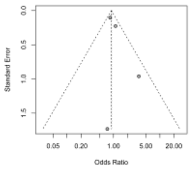

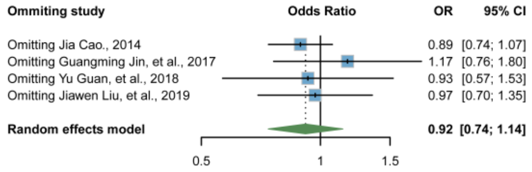

Hypertension (yes vs no)				
AMD-10	Zhilan Bai, et al.	2005	2835	
AMD-35	Guangming Jin, et al.	2017	4881	0.92 (0.71, 1.20)
AMD-31	Yu Guan, et al.	2018	5947	
AMD-27	Jiawen Liu, et al.	2019	1202	

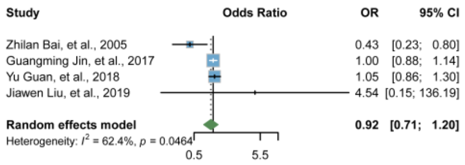

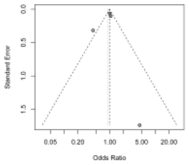

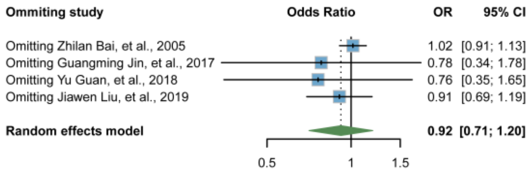

Notes: AMD, age-related macular degeneration; OR, odds ratio; CI, confidence interval.

Table S7. Detailed characteristics of included articles (n=40)

Study ID	Author (s)	Publication year	Province	Setting	Investigation year	Assessment	Grading system	Sample size	Female proportion	Any AMD	Early AMD	Late AMD	GA	NV AMD
AMD-01	Huang P, et al.	1992	Hunan	Mixed	1987	FI	CMA1986	1589	-	145	-	-	-	-
AMD-02	Tan JQ, et al.	1992	Hunan	Mixed	1989	FI	CMA1986	1040	0.2788	191	-	-	-	-
AMD-03	Wu ZQ, et al.	1992	Hunan	Mixed	1989	FI	CMA1986	3351	0.3286	197	-	-	-	-
AMD-04	Chen YH, et al.	1993	Shaanxi, Qinghai	Mixed	1991	FI	CMA1986	849	-	70	-	-	-	-
AMD-05	Yu Q, et al.	1994	Guangdong	Rural	1990	FI	CMA1986	932	0.5354	50	-	-	-	-
AMD-06	Xuan MZ, et al.	1994	Zhejiang	Mixed	1989	FI	CMA1986	730	0.5315	54	-	-	-	-
AMD-07	He MG, et al.	1998	Guangdong	Rural	1997	FI	CMA1986	5342	0.5468	451	-	-	-	-
AMD-08	Zou HD, et al.	2005	Shanghai	Urban	2003	FI	CMA1986	1023	0.5513	159	-	-	-	-
AMD-09	Tian MN, et al.	2005	Gansu	Mixed	2002	FI	CMA1986	7563	0.4542	579	-	-	-	-
AMD-10	Bai ZL, et al.	2005	Shaanxi	Rural	2003	FI	IC	2835	0.5605	85	-	-	-	-
AMD-11	Shi K, et al.	2009	Qinghai	Rural	2006	FI	CMA1986	2112	0.4673	132	-	-	-	-
AMD-12	Li HL, et al.	2009	Chongqing	Urban	2009	FI	"Ophthalmology" (7th version)	1513	0.8189	122	-	-	-	-
AMD-13	Zhao X, et al.	2011	Beijing	Urban	2006	FI	CARMS	2348	0.5281	77	70	7	3	4
AMD-14	Huang XB, et al.	2012	Shanghai	Urban	2008	FI	CMA1986	3571	0.5578	477	-	-	-	-
AMD-15	Yang Z.	2014	Sichuan	Urban	2013	FI	CARMS	2097	0.6433	207	194	13	9	4
AMD-16	Cao J.	2014	Jiangsu	Urban	2010	FI	CMA1986	6150	0.5751	482	-	-	-	-
AMD-17	Xiang W, et al.	2015	Ningxia	Rural	2014	FI	AREDS	4812	0.4994	54	46	8	-	-
AMD-18	Wang Y, et al.	2015	Jiangsu	Rural	2015	FI	CARMS	2985	0.6549	97	71	26	17	9
AMD-19	Li WL, et al.	2015	Ningxia	Rural	2013	FI	CARMS	679	0.4654	25	17	8	-	-
AMD-20	Li Y, et al.	2006	Beijing	Mixed	2001	FI	WARM	4376	0.5643	70	63	7	1	6
AMD-21	Chen SJ, et al.	2008	Taiwan	Mixed	1999	FI	WARM	1058	0.3771	117	97	20	1	19

Study ID	Author (s)	Publication year	Province	Setting	Investigation year	Assessment	Grading system	Sample size	Female proportion	Any AMD	Early AMD	Late AMD	GA	NV AMD
AMD-22	Huang TL, et al.	2010	Taiwan	Rural	2007	FI	WARM	2316	0.6002	121	-	-	-	-
AMD-23	Yang K, et al.	2011	Hebei	Rural	2006	FI	WARM	6581	0.5347	204	200	4	-	-
AMD-24	Ye H, et al.	2014	Shanghai	Urban	2013	FI	WARM	2005	0.5626	229	206	23	-	-
AMD-25	Huang EJ, et al.	2014	Taiwan	Mixed	2011	FI	WARM	673	0.4577	150	101	49	-	-
AMD-26	Wang H, et al.	2018	Chongqing	Rural	2015	FI	CARMS	2122	0.6517	200	-	-	-	-
AMD-27	Liu JW, et al.	2019	Xinjiang	Rural	2017	FI	Beckmann	1202	0.5308	73	48	25	12	13
AMD-28	Bai QX, et al.	2023	Beijing	Mixed	2018	FI	Beckmann	7719	0.5505	1607	-	-	-	-
AMD-29	Zhang YL, et al.	2020	Guangdong	Urban	2019	FI	Beckmann	942	0.7866	32	-	-	-	-
AMD-30	Mai JY, et al.	2017	Hainan	Mixed	2013	FI	Beckmann	675	0.5452	97	-	-	-	-
AMD-31	Guan Y, et al.	2018	Jiangsu	Rural	2011	FI	Beckmann	5947	0.5852	448	-	-	-	-
AMD-32	Xu HM, et al.	2018	Zhejiang	Urban	2015	FI	Beckmann	2363	0.5315	81	-	-	-	-
AMD-33	Zhang XY, et al.	2022	Qinghai	Rural	2019	FI	Beckmann	1223	0.2249	190	-	-	-	-
AMD-34	Guan RJ, et al.	2020	Qinghai	Urban	2017	FI	Beckmann	2595	0.5337	243	-	-	-	-
AMD-35	Jin GM, et al.	2017	Guangdong	Rural	2014	FI	Beckmann	4881	0.5087	2924	2882	42	-	-
AMD-36	Zhang KY, et al.	2017	Hainan	Mixed	2015	FI	Beckmann	2232	0.6564	357	331	26	-	-
AMD-37	Wang HJ, et al.	2017	Beijing	Mixed	2016	FI	CMA1986	1127	0.5146	19	-	-	-	-
AMD-38	Xi C, et al.	2017	Beijing	Rural	2010	FI	IC	242	0.5857	11	-	-	-	-
AMD-39	Lin YH, et al.	2022	Hunan	Mixed	2018	FI	WARM	43821	0.3966	1229	-	-	-	-
AMD-40	Li Y, et al.	2023	Xinjiang	Mixed	2020	FI	Beckmann	2529	0.5366	154	-	-	-	-

Notes: "-" represents unavailable data. AMD, age-related macular degeneration; GA, geographic atrophy; NVAMD, neovascular AMD; FI, Fundus imaging; CMA 1986, the "Age-related Macular Degeneration Clinical Diagnosis Standard" proposed by the China Medical Association in 1986; IC, the International Classification and Grading system; CARMS, the Clinical Age-Related Maculopathy Grading System; AREDS, the grading system proposed by the Age-Related Eye Disease Study Research Group; WARM, the Wisconsin age-related maculopathy system.

Table S8. Quality scores for assessing the risk of bias in the included studies (n=40)

Study ID	Author (s)	Publication year	1. Was the sample frame appropriate to address the target population?	2. Were study participants sampled in an appropriate way?	3. Was the sample size adequate?	4. Were the study subjects and the setting described in detail?	5. Was the data analysis conducted with sufficient coverage of the identified sample?	6. Were valid methods used for the identification of the condition?	7. Was the condition measured in a standard, reliable way for all participants?	8. Was there appropriate statistical analysis?	9. Was the response rate adequate, and if not, was the low response rate managed appropriately?	Total scores
AMD-01	Huang P, et al.	1992	1	1	1	0	1	1	1	0	1	7
AMD-02	Tan JQ, et al.	1992	1	0	1	1	1	1	0	0	0	5
AMD-03	Wu ZQ, et al.	1992	1	1	1	1	1	1	0	0	1	7
AMD-04	Chen YH, et al.	1993	1	1	1	0	1	1	0	0	0	5
AMD-05	Yu Q, et al.	1994	1	1	1	0	1	1	1	0	1	7
AMD-06	Xuan MZ, et al.	1994	1	1	1	1	1	1	0	0	1	7
AMD-07	He MG, et al.	1998	1	1	1	1	1	1	1	0	1	8
AMD-08	Zou HD, et al.	2005	1	1	1	1	1	1	1	1	1	9
AMD-09	Tian MN, et al.	2005	1	0	1	0	1	1	0	1	0	5
AMD-10	Bai ZL, et al.	2005	1	1	1	1	1	1	1	1	1	9
AMD-11	Shi K, et al.	2009	0	1	1	1	0	1	1	1	0	6
AMD-12	Li HL, et al.	2009	1	1	1	1	1	1	1	1	0	8
AMD-13	Zhao X, et al.	2011	1	1	1	1	1	1	1	1	1	9
AMD-14	Huang XB, et al.	2012	1	1	1	1	1	1	1	1	1	9
AMD-15	Yang Z.	2014	1	1	1	1	1	1	1	1	1	9
AMD-16	Cao J.	2014	1	1	1	1	1	1	1	1	1	9
AMD-17	Xiang W, et al.	2015	1	1	1	1	1	1	1	1	1	9
AMD-18	Wang Y, et al.	2015	1	1	1	1	1	1	0	1	1	8
AMD-19	Li WL, et al.	2015	1	1	1	1	1	1	0	1	0	7
AMD-20	Li Y, et al.	2006	1	1	1	1	1	1	1	1	1	9
AMD-21	Chen SJ, et al.	2008	1	1	1	1	1	1	0	1	1	8
AMD-22	Huang TL, et al.	2010	1	1	1	1	1	1	1	1	0	8
AMD-23	Yang K, et al.	2011	1	1	1	1	1	1	0	1	1	8
AMD-24	Ye H, et al.	2014	1	1	1	1	1	1	1	1	1	9
AMD-25	Huang EJ, et al.	2014	1	1	1	1	1	1	0	1	0	7
AMD-26	Wang H, et al.	2018	1	1	1	1	1	1	1	1	1	9
AMD-27	Liu JW, et al.	2019	1	1	1	1	1	1	1	1	1	9
AMD-28	Bai QX, et al.	2023	0	0	1	1	1	1	1	1	0	6
AMD-29	Zhang YL, et al.	2020	1	1	1	1	0	1	1	1	1	8
AMD-30	Mai JY, et al.	2017	0	0	1	1	1	1	0	1	0	5
AMD-31	Guan Y, et al.	2018	1	1	1	1	1	1	1	1	1	9
AMD-32	Xu HM, et al.	2018	1	1	1	1	1	1	1	1	1	9
AMD-33	Zhang XY, et al.	2022	1	1	1	0	0	1	1	1	0	6
AMD-34	Guan RJ, et al.	2020	0	1	1	1	0	1	0	1	1	6
AMD-35	Jin GM, et al.	2017	1	1	1	1	1	1	1	1	0	8
AMD-36	Zhang KY, et al.	2017	1	1	1	1	1	1	0	1	1	8
AMD-37	Wang HJ, et al.	2017	0	0	1	0	1	1	1	0	0	4

Study ID	Author (s)	Publication year	1. Was the sample frame appropriate to address the target population?	2. Were study participants sampled in an appropriate way?	3. Was the sample size adequate?	4. Were the study subjects and the setting described in detail?	5. Was the data analysis conducted with sufficient coverage of the identified sample?	6. Were valid methods used for the identification of the condition?	7. Was the condition measured in a standard, reliable way for all participants?	8. Was there appropriate statistical analysis?	9. Was the response rate adequate, and if not, was the low response rate managed appropriately?	Total scores
AMD-38	Xi C, et al.	2017	0	1	0	1	1	1	1	1	0	6
AMD-39	Lin YH, et al.	2022	0	0	1	1	1	1	1	1	0	6
AMD-40	Li Y, et al.	2023	0	0	1	1	1	1	0	1	0	5

Table S9. Summary of the main characteristics of the included studies (n=40)

Characteristics	Number of studies (%)
**Publication year**	
1991-2000	7 (17.5)
2001-2010	8 (20.0)
2011-2023	25 (62.5)
**Setting**	
Urban	10 (25.0)
Rural	15 (37.5)
Mixed	15 (37.5)
**Grading system**	
CMA 1986	13 (32.5)
IC	2 (5.0)
CARMS	5 (12.5)
AREDS	1 (2.5)
WARM	7 (17.5)
Beckmann	11 (27.5)
"Ophthalmology" (7th version)	1 (2.5)
**Sample size**	
< 2000	16 (40.0)
2001-4000	14 (35.0)
> 4000	10 (25.0)
**Study quality**	
High (7-9)	28 (70.0)
Moderate (4-6)	12 (30.0)

Notes: CMA 1986, the "Age-related Macular Degeneration Clinical Diagnosis Standard" proposed by the China Medical Association in 1986; IC, the International Classification and Grading system; CARMS, the Clinical Age-Related Maculopathy Grading System; AREDS, the grading system proposed by the Age-Related Eye Disease Study Research Group; WARM, the Wisconsin age-related maculopathy system.

Table S10. Sensitivity analysis of estimated prevalence (%) and case number (million) of any AMD in the Mainland of China in 2020 (additionally including studies with quality scores <7 or investigation year before 1990)

Age group (years)	Prevalence (%, 95% CI)			Case number (million, 95% CI)		
	Both	Male	Female	Both	Male	Female
40-49	2.43 (1.89-3.12)	2.63 (2.05-3.38)	2.23 (1.73-2.86)	5.04 (3.92-6.47)	2.79 (2.17-3.57)	2.26 (1.76-2.90)
50-59	3.86 (3.03-4.91)	4.18 (3.28-5.31)	3.55 (2.78-4.51)	8.60 (6.75-10.93)	4.68 (3.67-5.94)	3.92 (3.08-4.99)
60-69	6.42 (5.08-8.08)	6.93 (5.49-8.72)	5.92 (4.68-7.46)	9.47 (7.49-11.92)	5.07 (4.02-6.38)	4.39 (3.47-5.53)
70-79	9.93 (7.92-12.38)	10.71 (8.55-13.33)	9.21 (7.34-11.51)	8.03 (6.40-10.01)	4.17 (3.33-5.19)	3.86 (3.08-4.82)
80-89	15.28 (12.30-18.82)	16.45 (13.28-20.21)	14.37 (11.55-17.74)	4.77 (3.84-5.87)	2.24 (1.80-2.75)	2.53 (2.04-3.13)
40-89	5.21 (4.12-6.56)	5.51 (4.36-6.94)	4.91 (3.88-6.18)	35.90 (28.40-45.20)	18.94 (14.99-23.83)	16.97 (13.42-21.37)

Notes: AMD, age-related macular degeneration; CI, confidence interval.

Table S11. Estimated prevalence (%) and case number (million) of any AMD by geographical regions in the Mainland of China in 2020

Age group (years)	Prevalence (%, 95% CI)			Case number (million, 95% CI)		
	Both	Male	Female	Both	Male	Female
**North China**						
40-49	1.42 (0.93, 2.14)	1.45 (0.95, 2.19)	1.39 (0.92, 2.09)	0.34 (0.23, 0.52)	0.18 (0.12, 0.27)	0.16 (0.11, 0.25)
50-59	2.30 (1.52, 3.44)	2.35 (1.55, 3.53)	2.24 (1.48, 3.36)	0.61 (0.40, 0.92)	0.32 (0.21, 0.47)	0.30 (0.20, 0.44)
60-69	3.80 (2.53, 5.64)	3.89 (2.58, 5.78)	3.71 (2.47, 5.50)	0.76 (0.51, 1.13)	0.39 (0.26, 0.57)	0.38 (0.25, 0.56)
70-79	5.81 (3.87, 8.58)	5.91 (3.93, 8.75)	5.71 (3.81, 8.43)	0.55 (0.37, 0.81)	0.27 (0.18, 0.39)	0.28 (0.19, 0.42)
80-89	8.94 (6.00, 13.07)	9.11 (6.11, 13.32)	8.80 (5.91, 12.88)	0.31 (0.21, 0.46)	0.14 (0.09, 0.21)	0.17 (0.12, 0.25)
40-89	3.08 (2.04, 4.58)	3.08 (2.04, 4.59)	3.07 (2.04, 4.56)	2.58 (1.71, 3.84)	1.29 (0.85, 1.92)	1.29 (0.86, 1.92)
**Northeast China**						
40-49	1.12 (0.62, 2.01)	1.13 (0.63, 2.02)	1.12 (0.62, 2.01)	0.18 (0.10, 0.33)	0.09 (0.05, 0.17)	0.09 (0.05, 0.16)
50-59	1.83 (1.02, 3.27)	1.86 (1.03, 3.29)	1.81 (1.00, 3.24)	0.35 (0.19, 0.62)	0.18 (0.10, 0.31)	0.17 (0.10, 0.31)
60-69	3.07 (1.72, 5.39)	3.13 (1.76, 5.49)	3.01 (1.68, 5.31)	0.45 (0.25, 0.79)	0.22 (0.12, 0.39)	0.23 (0.13, 0.40)
70-79	4.73 (2.66, 8.28)	4.81 (2.71, 8.37)	4.67 (2.61, 8.20)	0.31 (0.17, 0.54)	0.14 (0.08, 0.25)	0.16 (0.09, 0.29)
80-89	7.38 (4.19, 12.68)	7.53 (4.30, 12.84)	7.27 (4.11, 12.55)	0.18 (0.10, 0.30)	0.08 (0.04, 0.13)	0.10 (0.06, 0.17)
40-89	2.49 (1.39, 4.38)	2.47 (1.39, 4.33)	2.50 (1.40, 4.43)	1.47 (0.82, 2.59)	0.72 (0.40, 1.26)	0.75 (0.42, 1.33)
**East China**						
40-49	2.26 (1.69, 2.99)	2.45 (1.84, 3.26)	2.05 (1.54, 2.71)	1.41 (1.05, 1.87)	0.78 (0.58, 1.03)	0.63 (0.47, 0.83)
50-59	3.53 (2.66, 4.64)	3.84 (2.89, 5.05)	3.21 (2.42, 4.23)	2.41 (1.82, 3.18)	1.32 (0.99, 1.73)	1.10 (0.83, 1.44)
60-69	5.83 (4.43, 7.61)	6.34 (4.81, 8.27)	5.33 (4.05, 6.94)	2.67 (2.02, 3.48)	1.44 (1.10, 1.89)	1.22 (0.93, 1.59)
70-79	8.66 (6.59, 11.25)	9.36 (7.12, 12.17)	8.00 (6.09, 10.38)	2.23 (1.69, 2.89)	1.17 (0.89, 1.52)	1.06 (0.81, 1.37)
80-89	13.12 (10.09, 16.82)	14.26 (10.98, 18.24)	12.25 (9.41, 15.73)	1.35 (1.04, 1.73)	0.64 (0.49, 0.81)	0.72 (0.55, 0.92)
40-89	4.73 (3.59, 6.18)	5.05 (3.83, 6.60)	4.42 (3.35, 5.77)	10.07 (7.63, 13.15)	5.34 (4.05, 6.98)	4.72 (3.58, 6.16)
**South Central China**						
40-49	2.90 (2.00, 4.16)	3.17 (2.19, 4.56)	2.60 (1.80, 3.73)	1.66 (1.15, 2.39)	0.94 (0.65, 1.35)	0.73 (0.50, 1.04)

Age group (years)	Prevalence (%, 95% CI)			Case number (million, 95% CI)		
	Both	Male	Female	Both	Male	Female
50-59	4.47 (3.14, 6.29)	4.92 (3.46, 6.93)	4.01 (2.82, 5.64)	2.70 (1.90, 3.80)	1.50 (1.05, 2.11)	1.20 (0.85, 1.69)
60-69	7.24 (5.18, 10.01)	7.94 (5.67, 10.96)	6.56 (4.69, 9.06)	2.73 (1.95, 3.77)	1.49 (1.06, 2.05)	1.24 (0.89, 1.72)
70-79	10.64 (7.68, 14.51)	11.59 (8.36, 15.78)	9.75 (7.03, 13.31)	2.24 (1.61, 3.05)	1.18 (0.85, 1.61)	1.06 (0.76, 1.44)
80-89	16.06 (11.71, 21.51)	17.60 (12.88, 23.47)	14.92 (10.85, 20.06)	1.35 (0.98, 1.80)	0.63 (0.46, 0.84)	0.72 (0.52, 0.97)
40-89	5.77 (4.11, 8.01)	6.20 (4.40, 8.60)	5.35 (3.81, 7.41)	10.68 (7.60, 14.82)	5.73 (4.07, 7.95)	4.95 (3.52, 6.86)
**Southwest China**						
40-49	2.89 (2.13, 3.88)	3.30 (2.43, 4.45)	2.46 (1.82, 3.29)	0.90 (0.66, 1.21)	0.53 (0.39, 0.71)	0.37 (0.28, 0.50)
50-59	4.47 (3.33, 5.94)	5.11 (3.80, 6.81)	3.81 (2.85, 5.06)	1.42 (1.06, 1.89)	0.82 (0.61, 1.09)	0.61 (0.45, 0.80)
60-69	7.43 (5.61, 9.72)	8.48 (6.40, 11.12)	6.38 (4.83, 8.33)	1.49 (1.13, 1.95)	0.85 (0.64, 1.11)	0.64 (0.49, 0.84)
70-79	10.94 (8.31, 14.22)	12.45 (9.44, 16.19)	9.51 (7.23, 12.35)	1.41 (1.07, 1.84)	0.78 (0.59, 1.02)	0.63 (0.48, 0.82)
80-89	16.08 (12.36, 20.58)	18.47 (14.20, 23.61)	14.15 (10.86, 18.13)	0.77 (0.59, 0.98)	0.39 (0.30, 0.50)	0.37 (0.29, 0.48)
40-89	5.95 (4.48, 7.81)	6.69 (5.03, 8.80)	5.21 (3.93, 6.83)	6.00 (4.52, 7.88)	3.37 (2.53, 4.44)	2.63 (1.98, 3.44)
**Northwest China**						
40-49	1.60 (1.12, 2.31)	1.71 (1.20, 2.46)	1.49 (1.03, 2.15)	0.25 (0.17, 0.36)	0.14 (0.10, 0.20)	0.11 (0.08, 0.16)
50-59	2.61 (1.84, 3.70)	2.80 (1.98, 3.97)	2.40 (1.69, 3.43)	0.42 (0.29, 0.59)	0.23 (0.16, 0.32)	0.19 (0.13, 0.27)
60-69	4.49 (3.22, 6.23)	4.86 (3.49, 6.73)	4.11 (2.95, 5.72)	0.42 (0.30, 0.58)	0.23 (0.16, 0.31)	0.19 (0.14, 0.26)
70-79	6.84 (4.90, 9.49)	7.41 (5.32, 10.25)	6.31 (4.51, 8.81)	0.35 (0.25, 0.49)	0.18 (0.13, 0.25)	0.17 (0.12, 0.24)
80-89	10.20 (7.36, 14.03)	10.99 (7.94, 15.10)	9.53 (6.87, 13.13)	0.19 (0.13, 0.26)	0.09 (0.07, 0.13)	0.09 (0.07, 0.13)
40-89	3.39 (2.41, 4.76)	3.60 (2.56, 5.04)	3.19 (2.26, 4.48)	1.62 (1.15, 2.28)	0.87 (0.62, 1.21)	0.76 (0.54, 1.06)
**Total China**						
40-49	2.29 (1.63, 3.22)	2.51 (1.78, 3.52)	2.07 (1.47, 2.91)	4.75 (3.37, 6.67)	2.65 (1.88, 3.72)	2.10 (1.49, 2.95)
50-59	3.56 (2.55, 4.95)	3.89 (2.79, 5.40)	3.22 (2.31, 4.48)	7.92 (5.67, 11.01)	4.35 (3.12, 6.05)	3.56 (2.55, 4.96)
60-69	5.78 (4.18, 7.93)	6.30 (4.57, 8.64)	5.26 (3.80, 7.23)	8.51 (6.16, 11.70)	4.62 (3.34, 6.33)	3.90 (2.82, 5.37)
70-79	8.77 (6.40, 11.90)	9.57 (7.00, 12.95)	8.03 (5.85, 10.93)	7.09 (5.17, 9.62)	3.72 (2.72, 5.04)	3.37 (2.45, 4.58)

Age group (years)	Prevalence (%, 95% CI)			Case number (million, 95% CI)		
	**Both**	**Male**	**Female**	**Both**	**Male**	**Female**
80-89	13.28 (9.80, 17.75)	14.49 (10.73, 19.29)	12.35 (9.08, 16.57)	4.15 (3.06, 5.54)	1.97 (1.46, 2.62)	2.18 (1.60, 2.92)
40-89	4.70 (3.40, 6.46)	5.04 (3.65, 6.92)	4.37 (3.15, 6.01)	32.42 (23.43, 44.54)	17.31 (12.53, 23.76)	15.10 (10.91, 20.78)

Notes: AMD, age-related macular degeneration; CI, confidence interval.

Table S12. Estimated prevalence (%) and case number (million) of any AMD by economic regions in the Mainland of China in 2020

Age group (years)	Prevalence (%, 95%CI)			Case number (million, 95%CI)		
	Both	Male	Female	Both	Male	Female
**East China**						
40-49	2.44 (1.71, 3.47)	2.67 (1.87, 3.80)	2.20 (1.54, 3.12)	2.00 (1.40, 2.84)	1.12 (0.79, 1.60)	0.89 (0.60, 1.31)
50-59	3.74 (2.65, 5.23)	4.08 (2.89, 5.72)	3.38 (2.40, 4.74)	3.20 (2.27, 4.48)	1.77 (1.25, 2.48)	1.42 (0.98, 2.06)
60-69	5.92 (4.25, 8.16)	6.43 (4.62, 8.86)	5.42 (3.89, 7.47)	3.48 (2.50, 4.79)	1.87 (1.35, 2.58)	1.56 (1.08, 2.22)
70-79	8.84 (6.39, 12.06)	9.59 (6.94, 13.06)	8.15 (5.89, 11.13)	2.69 (1.94, 3.67)	1.40 (1.01, 1.91)	1.26 (0.89, 1.77)
80-89	13.59 (9.96, 18.21)	14.77 (10.85, 19.70)	12.71 (9.28, 17.09)	1.68 (1.23, 2.25)	0.78 (0.58, 1.04)	0.89 (0.64, 1.23)
40-89	4.85 (3.47, 6.70)	5.17 (3.70, 7.15)	4.53 (3.24, 6.25)	13.04 (9.34, 18.02)	6.95 (4.97, 9.61)	6.25 (4.36, 8.91)
**Central China**						
40-49	2.21 (1.66, 2.92)	2.39 (1.79, 3.16)	2.02 (1.52, 2.67)	1.13 (0.85, 1.49)	0.62 (0.46, 0.82)	0.51 (0.38, 0.68)
50-59	3.52 (2.65, 4.62)	3.83 (2.89, 5.03)	3.21 (2.43, 4.21)	2.06 (1.56, 2.71)	1.11 (0.84, 1.46)	0.95 (0.72, 1.24)
60-69	5.86 (4.45, 7.63)	6.38 (4.84, 8.31)	5.34 (4.07, 6.95)	2.19 (1.66, 2.85)	1.19 (0.90, 1.55)	1.00 (0.76, 1.30)
70-79	8.76 (6.68, 11.35)	9.48 (7.21, 12.30)	8.08 (6.16, 10.45)	1.92 (1.46, 2.49)	1.01 (0.77, 1.32)	0.91 (0.69, 1.18)
80-89	13.13 (10.11, 16.81)	14.29 (11.00, 18.29)	12.24 (9.42, 15.69)	1.08 (0.83, 1.39)	0.51 (0.39, 0.65)	0.57 (0.44, 0.73)
40-89	4.73 (3.59, 6.16)	5.06 (3.84, 6.60)	4.41 (3.35, 5.73)	8.38 (6.36, 10.92)	4.44 (3.37, 5.80)	3.94 (2.99, 5.12)
**West China**						
40-49	2.49 (1.77, 3.48)	2.76 (1.96, 3.85)	2.20 (1.56, 3.09)	1.44 (1.02, 2.01)	0.82 (0.58, 1.14)	0.62 (0.44, 0.87)
50-59	3.90 (2.79, 5.40)	4.32 (3.10, 5.98)	3.46 (2.48, 4.80)	2.30 (1.65, 3.19)	1.29 (0.93, 1.79)	1.01 (0.72, 1.40)
60-69	6.53 (4.75, 8.90)	7.25 (5.28, 9.86)	5.81 (4.22, 7.93)	2.40 (1.75, 3.27)	1.33 (0.97, 1.81)	1.07 (0.78, 1.46)
70-79	9.89 (7.25, 13.31)	10.98 (8.08, 14.75)	8.87 (6.49, 11.98)	2.17 (1.59, 2.92)	1.16 (0.86, 1.56)	1.01 (0.74, 1.36)
80-89	14.69 (10.89, 19.49)	16.28 (12.12, 21.51)	13.41 (9.91, 17.87)	1.21 (0.90, 1.60)	0.60 (0.44, 0.79)	0.61 (0.45, 0.82)
40-89	5.18 (3.76, 7.07)	5.64 (4.10, 7.69)	4.71 (3.42, 6.45)	9.52 (6.91, 13.00)	5.20 (3.78, 7.10)	4.32 (3.13, 5.91)
**Northeast China**						
40-49	1.12 (0.62, 2.01)	1.13 (0.63, 2.02)	1.12 (0.62, 2.01)	0.18 (0.10, 0.33)	0.09 (0.05, 0.17)	0.09 (0.05, 0.16)

Age group (years)	Prevalence (%, 95%CI)			Case number (million, 95%CI)		
	Both	Male	Female	Both	Male	Female
50-59	1.83 (1.02, 3.27)	1.86 (1.03, 3.29)	1.81 (1.00, 3.24)	0.35 (0.19, 0.62)	0.18 (0.10, 0.31)	0.17 (0.10, 0.31)
60-69	3.07 (1.72, 5.39)	3.13 (1.76, 5.49)	3.01 (1.68, 5.31)	0.45 (0.25, 0.79)	0.22 (0.12, 0.39)	0.23 (0.13, 0.40)
70-79	4.73 (2.66, 8.28)	4.81 (2.71, 8.37)	4.67 (2.61, 8.20)	0.31 (0.17, 0.54)	0.14 (0.08, 0.25)	0.16 (0.09, 0.29)
80-89	7.38 (4.19, 12.68)	7.53 (4.30, 12.84)	7.27 (4.11, 12.55)	0.18 (0.10, 0.30)	0.08 (0.04, 0.13)	0.10 (0.06, 0.17)
40-89	2.49 (1.39, 4.38)	2.47 (1.39, 4.33)	2.50 (1.40, 4.43)	1.47 (0.82, 2.59)	0.72 (0.40, 1.26)	0.75 (0.42, 1.33)
**Total China**						
40-49	2.29 (1.63, 3.22)	2.51 (1.78, 3.52)	2.07 (1.47, 2.91)	4.75 (3.37, 6.67)	2.65 (1.88, 3.72)	2.10 (1.49, 2.95)
50-59	3.56 (2.55, 4.95)	3.89 (2.79, 5.40)	3.22 (2.31, 4.48)	7.92 (5.67, 11.01)	4.35 (3.12, 6.05)	3.56 (2.55, 4.96)
60-69	5.78 (4.18, 7.93)	6.30 (4.57, 8.64)	5.26 (3.80, 7.23)	8.51 (6.16, 11.70)	4.62 (3.34, 6.33)	3.90 (2.82, 5.37)
70-79	8.77 (6.40, 11.90)	9.57 (7.00, 12.95)	8.03 (5.85, 10.93)	7.09 (5.17, 9.62)	3.72 (2.72, 5.04)	3.37 (2.45, 4.58)
80-89	13.28 (9.80, 17.75)	14.49 (10.73, 19.29)	12.35 (9.08, 16.57)	4.15 (3.06, 5.54)	1.97 (1.46, 2.62)	2.18 (1.60, 2.92)
40-89	4.70 (3.40, 6.46)	5.04 (3.65, 6.92)	4.37 (3.15, 6.01)	32.42 (23.43, 44.54)	17.31 (12.53, 23.76)	15.10 (10.91, 20.78)

Notes: AMD, age-related macular degeneration; CI, confidence interval.

Table S13. Estimated provincial prevalence (%) and case number (million) of any AMD in the Mainland of China in 2020

Province	Prevalence (%, 95% CI)			Case number (million, 95% CI)		
	Both	Male	Female	Both	Male	Female
**Beijing**	2.88 (1.83, 4.45)	2.74 (1.74, 4.24)	3.02 (1.92, 4.66)	0.31 (0.20, 0.48)	0.15 (0.09, 0.23)	0.16 (0.10, 0.25)
**Tianjin**	3.05 (2.00, 4.59)	2.79 (1.83, 4.21)	3.31 (2.17, 4.97)	0.21 (0.14, 0.32)	0.10 (0.06, 0.15)	0.12 (0.08, 0.17)
**Hebei**	3.11 (2.12, 4.51)	3.06 (2.08, 4.45)	3.16 (2.15, 4.58)	1.11 (0.75, 1.61)	0.54 (0.36, 0.78)	0.57 (0.39, 0.83)
**Shanxi**	3.25 (2.22, 4.69)	3.42 (2.34, 4.94)	3.07 (2.10, 4.42)	0.56 (0.39, 0.81)	0.30 (0.21, 0.43)	0.26 (0.18, 0.38)
**Inner Mongolia**	2.94 (1.81, 4.70)	3.13 (1.92, 5.01)	2.75 (1.70, 4.40)	0.39 (0.24, 0.62)	0.21 (0.13, 0.33)	0.18 (0.11, 0.29)
**Liaoning**	2.99 (1.77, 4.95)	3.18 (1.89, 5.28)	2.80 (1.66, 4.64)	0.76 (0.45, 1.26)	0.40 (0.24, 0.66)	0.36 (0.22, 0.60)
**Jilin**	2.28 (1.24, 4.10)	2.07 (1.13, 3.72)	2.49 (1.36, 4.46)	0.32 (0.18, 0.58)	0.14 (0.08, 0.26)	0.18 (0.10, 0.32)
**Heilongjiang**	1.98 (1.00, 3.83)	1.83 (0.92, 3.54)	2.12 (1.07, 4.12)	0.38 (0.19, 0.74)	0.17 (0.09, 0.34)	0.21 (0.11, 0.40)
**Shanghai**	5.02 (3.92, 6.37)	5.07 (3.95, 6.44)	4.97 (3.88, 6.29)	0.64 (0.50, 0.82)	0.33 (0.26, 0.42)	0.31 (0.25, 0.40)
**Jiangsu**	4.92 (3.82, 6.26)	5.32 (4.13, 6.78)	4.53 (3.52, 5.75)	2.19 (1.70, 2.78)	1.17 (0.90, 1.49)	1.02 (0.79, 1.30)
**Zhejiang**	5.05 (3.93, 6.43)	5.47 (4.25, 6.96)	4.62 (3.60, 5.87)	1.67 (1.30, 2.13)	0.92 (0.72, 1.17)	0.75 (0.58, 0.95)
**Anhui**	4.46 (3.47, 5.68)	4.56 (3.54, 5.80)	4.38 (3.41, 5.56)	1.35 (1.05, 1.72)	0.69 (0.53, 0.87)	0.67 (0.52, 0.85)
**Fujian**	6.99 (5.07, 9.47)	8.38 (6.08, 11.37)	5.58 (4.06, 7.56)	1.34 (0.97, 1.81)	0.80 (0.58, 1.09)	0.53 (0.39, 0.72)
**Jiangxi**	5.40 (4.14, 6.97)	5.82 (4.45, 7.52)	4.99 (3.83, 6.43)	1.13 (0.87, 1.46)	0.61 (0.47, 0.79)	0.52 (0.40, 0.68)
**Shandong**	3.36 (2.39, 4.67)	3.26 (2.31, 4.54)	3.45 (2.46, 4.80)	1.74 (1.24, 2.42)	0.83 (0.59, 1.16)	0.91 (0.65, 1.27)
**Henan**	3.95 (2.95, 5.24)	4.19 (3.12, 5.57)	3.72 (2.78, 4.93)	1.78 (1.33, 2.36)	0.91 (0.68, 1.21)	0.87 (0.65, 1.15)
**Hubei**	5.12 (3.99, 6.50)	5.48 (4.26, 6.97)	4.75 (3.71, 6.03)	1.54 (1.20, 1.96)	0.83 (0.64, 1.05)	0.72 (0.56, 0.91)
**Hunan**	6.02 (4.58, 7.82)	6.65 (5.06, 8.64)	5.39 (4.11, 6.99)	2.01 (1.53, 2.61)	1.11 (0.84, 1.44)	0.90 (0.68, 1.16)
**Guangdong**	7.01 (4.68, 10.27)	7.66 (5.11, 11.22)	6.33 (4.23, 9.27)	3.50 (2.34, 5.13)	1.95 (1.30, 2.86)	1.55 (1.04, 2.27)
**Guangxi**	6.84 (4.53, 10.07)	6.78 (4.49, 9.99)	6.90 (4.57, 10.15)	1.52 (1.00, 2.23)	0.76 (0.50, 1.12)	0.76 (0.50, 1.12)
**Hainan**	7.64 (4.61, 12.22)	7.53 (4.54, 12.03)	7.76 (4.68, 12.42)	0.33 (0.20, 0.52)	0.17 (0.10, 0.26)	0.16 (0.10, 0.26)
**Chongqing**	4.70 (3.64, 6.00)	4.28 (3.31, 5.47)	5.11 (3.97, 6.52)	0.79 (0.61, 1.01)	0.36 (0.28, 0.46)	0.43 (0.34, 0.55)
**Sichuan**	5.82 (4.54, 7.39)	6.85 (5.33, 8.70)	4.81 (3.75, 6.09)	2.59 (2.02, 3.29)	1.52 (1.18, 1.93)	1.08 (0.84, 1.36)

Province	Prevalence (%, 95% CI)			Case number (million, 95% CI)		
	Both	Male	Female	Both	Male	Female
**Guizhou**	6.74 (4.96, 9.04)	7.73 (5.68, 10.38)	5.76 (4.24, 7.71)	1.12 (0.82, 1.50)	0.64 (0.47, 0.86)	0.48 (0.35, 0.64)
**Yunnan**	6.72 (4.75, 9.35)	7.65 (5.40, 10.65)	5.77 (4.08, 8.02)	1.45 (1.03, 2.02)	0.84 (0.59, 1.17)	0.61 (0.43, 0.85)
**Tibet**	3.60 (2.79, 4.59)	3.12 (2.41, 3.99)	4.12 (3.20, 5.24)	0.04 (0.03, 0.06)	0.02 (0.02, 0.03)	0.02 (0.02, 0.03)
**Shaanxi**	4.12 (3.10, 5.40)	4.43 (3.33, 5.82)	3.80 (2.87, 4.98)	0.79 (0.60, 1.04)	0.43 (0.32, 0.56)	0.36 (0.27, 0.48)
**Gansu**	3.58 (2.58, 4.90)	3.83 (2.76, 5.25)	3.33 (2.40, 4.55)	0.44 (0.32, 0.60)	0.24 (0.17, 0.32)	0.20 (0.15, 0.28)
**Qinghai**	3.25 (2.31, 4.52)	3.49 (2.48, 4.86)	3.01 (2.14, 4.17)	0.08 (0.06, 0.12)	0.05 (0.03, 0.06)	0.04 (0.03, 0.05)
**Ningxia**	3.22 (2.16, 4.75)	3.68 (2.46, 5.43)	2.75 (1.84, 4.05)	0.10 (0.07, 0.15)	0.06 (0.04, 0.09)	0.04 (0.03, 0.06)
**Xinjiang**	1.96 (1.07, 3.51)	1.86 (1.02, 3.33)	2.07 (1.13, 3.71)	0.21 (0.11, 0.37)	0.10 (0.06, 0.18)	0.11 (0.06, 0.19)

Notes: AMD, age-related macular degeneration; CI, confidence interval.

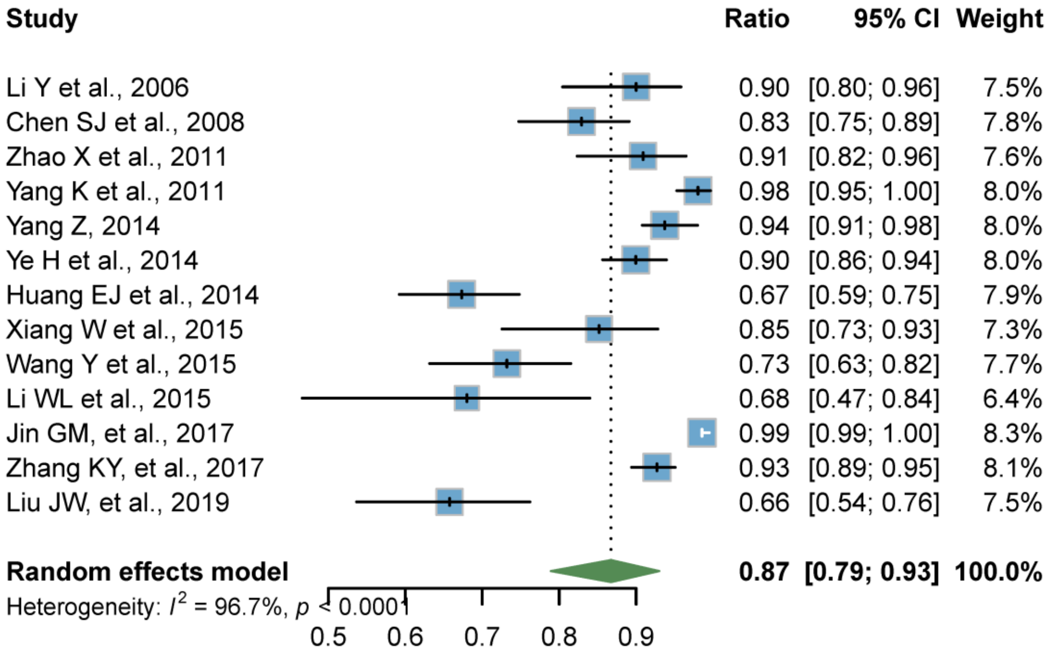

Figure S1. Meta-analysis of the early AMD to any AMD ratio for subtype imputation

Notes: AMD, age-related macular degeneration; CI, confidence interval.

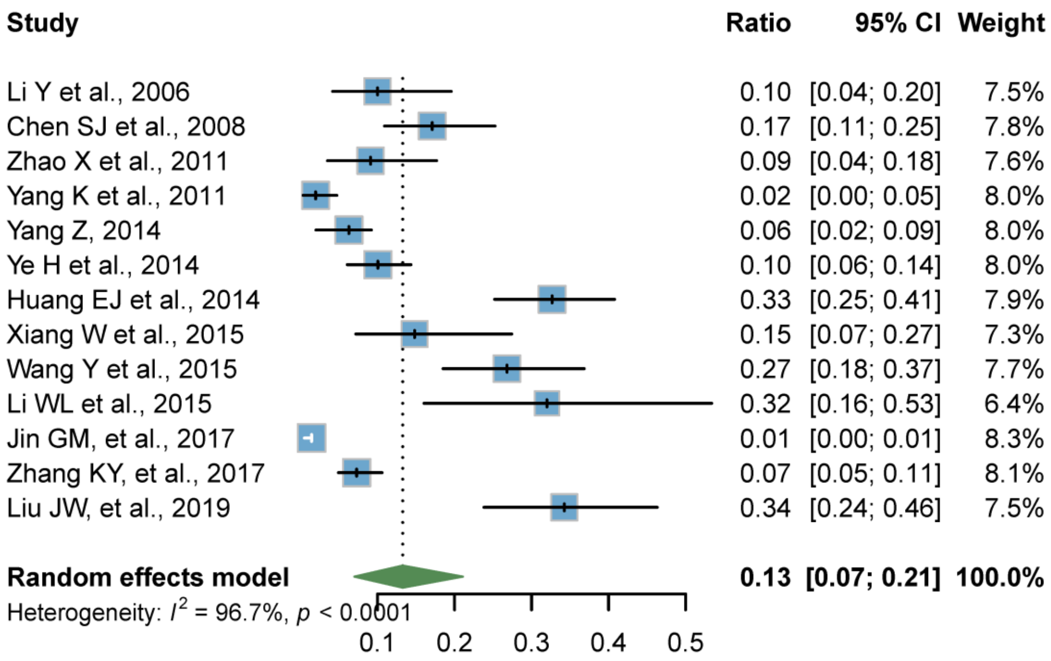

Figure S2. Meta-analysis of the late AMD to any AMD ratio for subtype imputation

Notes: AMD, age-related macular degeneration; CI, confidence interval.

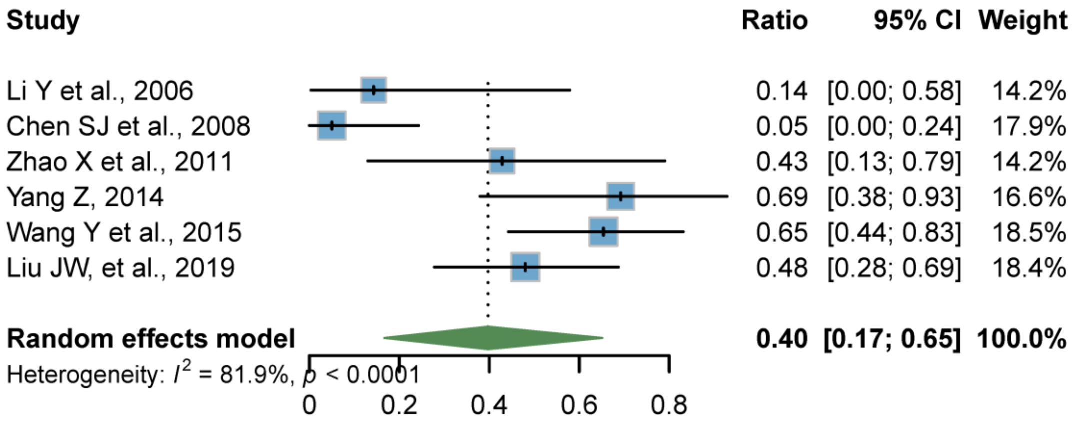

Figure S3. Meta-analysis of the GA to late AMD ratio for subtype imputation

Notes: AMD, age-related macular degeneration; GA, geographic atrophy; CI, confidence interval.

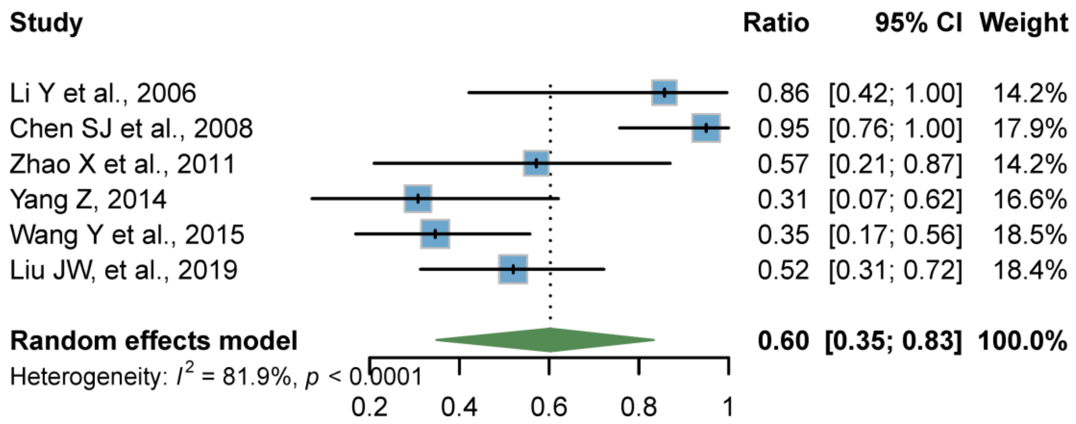

Figure S4. Meta-analysis of the NVAMD to late AMD ratio for subtype imputation

Notes: AMD, age-related macular degeneration; NVAMD, neovascular AMD; CI, confidence interval.

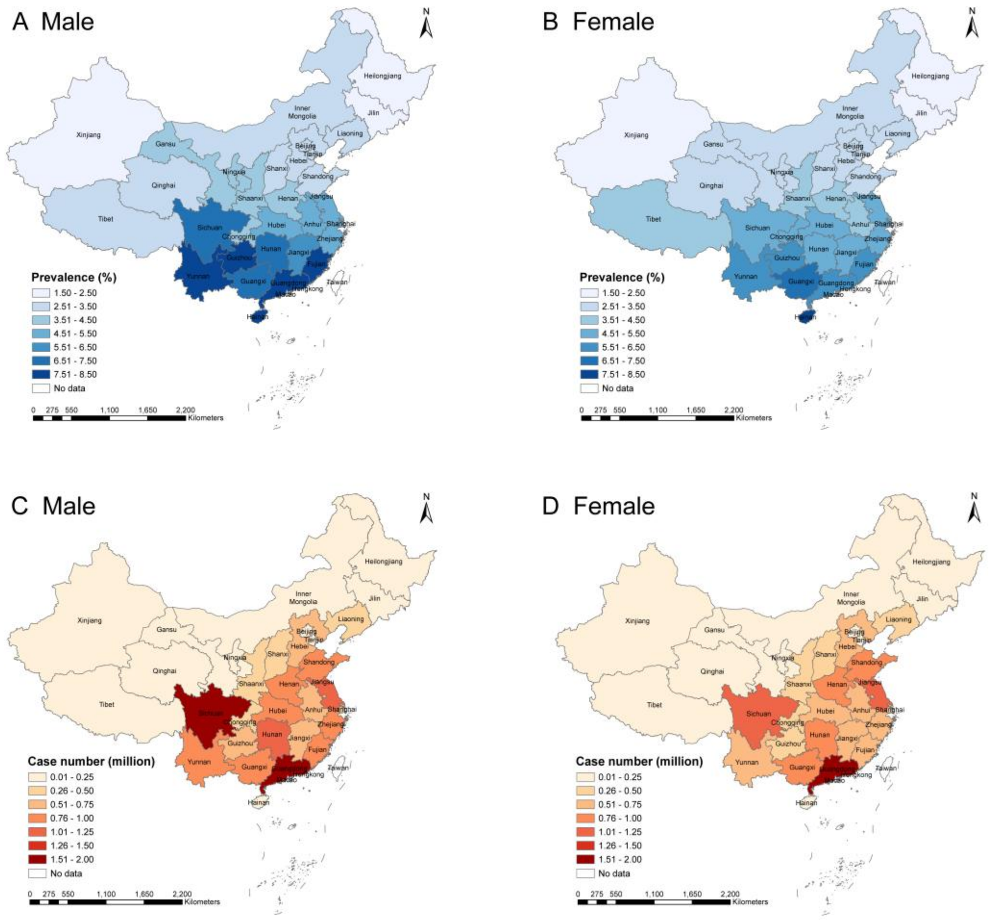

Figure S5. Estimated provincial prevalence (%) and case number (million) of any AMD among individuals aged 40-89 years in the Mainland of China in 2020

Notes: AMD, age-related macular degeneration.

Appendix 4. Full list of the included articles (n=40)

Study ID	Reference
AMD-01	Ping Huang, Ren-xiu He, Guo-zhen He, et al. 黄平，何仁秀，何国桢，等. The investigation of Age-related Macular Degeneration in Hunan Province* (湖南省老年黄斑变性流行病学调查) [J]. Chinese Ophthal Res (眼科研究). 1992(01): 60-61.
AMD-02	Jia-quan Tan, Ai-guang Nie, De-yong Jiang, et al. 谭家铨，聂爱光，姜德泳，等. The investigation of Age-related Macular Degeneration* (老年性黄斑变性流行病学调查) [J]. Hunan Medical Journal (湖南医学). 1992(05): 274-275.
AMD-03	Zheng-qing Wu, Jin-e Cao, Xiu-heng Yao, et al. 武正清，曹金娥，姚秀衡，等. Epidemiologic survey of senile macular degeneration (老年黄斑变性的流行病学调查) [J]. Chinese Journal of Ophthalmology (中华眼科杂志). 1992, 28(4): 246-247.
AMD-04	Yu-hua Chen, Ji-kui Shen, Yu-hong Zhang, et al. 陈玉华，申济奎，张宇弘，等. Senile macular degeneration in plateau aerea (高原地区老年性黄斑变性) [J]. Journal of High Altitude Medicine (实用眼科杂志). 1993(04): 57-58.
AMD-05	Qiang Yu, Jing-jing Xu, Si-ping Zhu, et al. 于强，许京京，朱斯平，等. Epidemiologic survey of Age-related Macular Degeneration in Doumen County, Guangdong province* (广东省斗门县老年黄斑变性流行病学调查) [J]. Chinese Journal of Ocular Fundus Diseases (中华眼底病杂志). 1994(2).
AMD-06	Meng-zheng Xuan, Jing Wang, Jin Zhao, et al. 宣梦铮，王竞，赵瑾，等. Epidemiologic survey of Age-related Macular Degeneration* (老年性黄斑变性的流行病学调查) [J]. Journal of Zhejiang Medical University (浙江医科大学学报). 1994(2).
AMD-07	Ming-guang He, Jing-jing Xu, Kai-li Wu, et al. 何明光，许京京，吴开力，等. The prevalence of age-related macular degeneration in Doumen county, Guangdong (广东省斗门县老年性黄斑变性流行病学调查) [J]. Chin J Ocul Fundus Dis (中华眼底病杂志). 1998(02): 61-63.
AMD-08	Hai-dong Zou, Zhe Zhang, Xun Xu, et al. 邹海东，张哲，许迅，等. Prevalence study of age-related macular degeneration in Caojiadu blocks, Shanghai (上海市静安区曹家渡街道年龄相关性黄斑变性的患病率调查) [J]. Chin J Ophthalmol (中华眼科杂志). 2005, 41(1): 15-19.
AMD-09	Man-nan Tian, Yue-mei Zhang, Li Li, et al. 田蔓男，张月梅，李丽，等. Epidemiologic survey of Age-related Macular Degeneration* (老年性黄斑变性的流行病学调查)[J]. Journal of Lanzhou University (Medical Sciences) (兰州大学学报（医学版）). 2005, 31(2): 70-71.
AMD-10	Zhi-lan Bai, Bai-chao Ren, Jian-gang Yang, et al. 白芝兰，任百超，杨建刚，等. Epidemiological investigation on age-related macular degeneration in rural area of Shaanxi Province, China (中国陕西省农村年龄相关性黄斑变性流行病学调查) [J]. International Journal of Ophthalmology (国际眼科杂志). 2005, 5(6): 1114-1121.
AMD-11	Kai Shi, Wen-fang Zhang, Xiao-yan Zhou, et al. 史凯，张文芳，周晓燕，等. Epidemiological investigation of ocular funds disease in Mongol above 40 years in Henan county (青海省河南县 40 岁以上世居蒙古族人群眼底病的流行病学调查) [J]. Chin Ophthal Res (眼科研究). 2009, 27(3): 239-242.
AMD-12	Hui-li Li, Ai-lin You, Di-ling Wan, et al. 李慧丽，犹爱林，万迪玲，等. Prevalence study of age-related macular degeneration in central urban area of Chongqing

Study ID	Reference
	(重庆市主城区年龄相关性黄斑变性患病率调查) [J]. Chin J Pract Ophthalmol (中国实用眼科杂志). 2009, 27(12): 1425-1429.
AMD-13	Xin Zhao, Bi-qi Tian, Yun-he Hao, et al. 赵欣, 田碧琪, 郝云赫, 等. Prevalence of age-related maculopathy in community of Xi Chang'an street of Beijing (北京西长安街社区 50 岁以上人群年龄相关性黄斑变性患病率调查) [J]. Int J Ophthalmol (国际眼科杂志). 2011, 11(8): 1364-1368.
AMD-14	Xiao-bo Huang, Hai-dong Zou, Ning Wang, et al. 黄晓波, 邹海东, 王宁, 等. Prevalence of age-related macular degeneration in Beixinjing Community of Shanghai (上海市北新泾街道老年人年龄相关性黄斑变性的患病率调查) [J]. Journal of Shanghai Jiaotong University (Medical Science) (上海交通大学学报(医学版)). 2012(02): 155-159.
AMD-15	Zhen Yang 杨桢. Prevalence and Associated Risk Factors of Age-Related Macular Degeneration in 50 years and older Population In ShunQing District, NanChong (南充市顺庆区 50 岁及以上人群年龄相关性黄斑变性患病率及相关因素分析) [D]. North Sichuan Medical University (川北医学院), 2014.
AMD-16	Jia Cao 曹葮. Prevalence study of age-related macular degeneration over the age of 50's in Wuxi (无锡市 50 岁及以上人群年龄相关性黄斑变性流行病学调查)[D]. Nanjing Medical University (南京医科大学), 2014.
AMD-17	Wei Xiang, Hui-ping Li, Yang Liu, et al. 向伟, 李慧平, 刘洋, 等. Prevalence Investigation of Age-related Macular Degeneration among Population Aged 50 Years or Above in Tongxin County of Ningxia (宁夏同心县≥50 岁人群年龄相关性黄斑变性患病率、危险因素及致盲情况分析) [J]. Journal of Ningxia Medical University (宁夏医科大学学报). 2015, 37(8): 927-930.
AMD-18	Ying Wang, Huai-jin Guan, Hong Lu, et al. 汪颖, 管怀进, 陆宏, 等. The Prevalence of age-related macular degeneration in the rural area of Qidong County, Jiangsu Province (江苏省启东市农村地区老年性黄斑变性流行病学调查分析) [J]. Chin J Ocul Fundus Dis (中华眼底病杂志). 2015, 31(5): 459-461.
AMD-19	Wu-liang Li, Hui-ping Li, Na Li, et al. 李武靓, 李慧平, 李娜, 等. Prevalence of age-related macular degeneration in elderly population in the rural area of Ningxia (宁夏农村地区 60 岁以上人群年龄相关性黄斑变性的患病率调查) [J]. Ningxia Medical Journal (宁夏医学杂志). 2015, 37(5): 401-404.
AMD-20	Li Y, Xu L, Jonas JB, et al. Prevalence of age-related maculopathy in the adult population in China: the Beijing eye study. AM J OPHTHALMOL 2006;142:788-793.
AMD-21	Chen SJ, Cheng CY, Peng KL, et al. Prevalence and associated risk factors of age-related macular degeneration in an elderly Chinese population in Taiwan: the Shihpai Eye Study. Invest Ophthalmol Vis Sci 2008;49:3126-3133.
AMD-22	Huang TL, Hsu SY, Tsai RK, Sheu MM. Etiology of ocular diseases in elderly Amis aborigines in Eastern Taiwan (The Amis Eye Study). JPN J OPHTHALMOL 2010;54:266-271.
AMD-23	Yang K, Liang YB, Gao LQ, et al. Prevalence of age-related macular degeneration in a rural Chinese population: the Handan Eye Study. OPHTHALMOLOGY 2011;118:1395-1401.

Study ID	Reference
AMD-24	Ye H, Zhang Q, Liu X, et al. Prevalence of age-related macular degeneration in an elderly urban Chinese population in China: the Jiangning Eye Study. Invest Ophthalmol Vis Sci 2014;55:6374-6380.
AMD-25	Huang EJ, Wu SH, Lai CH, et al. Prevalence and risk factors for age-related macular degeneration in the elderly Chinese population in south-western Taiwan: the Puzih eye study. Eye (Lond) 2014;28:705-714.
AMD-26	Hao Wang, Xi-yuan Zhou, Su Liu, et al. 王皓, 周希瑗, 刘苏, 等. Epidemiological investigation of age-related macular degeneration of over 50 years old Tuijia ethnic group in Chongqing rural district (重庆市农村地区 50 岁及以上土家族人群年龄相关性黄斑变性的流行病学调查) [J]. Chinese Journal of Ophthalmologic Medicine (Electronic Edition) (中华眼科医学杂志(电子版)). 2018,8(04): 157-162.
AMD-27	Jia-wen Liu, Bing-sheng Lou, Yao Li, et al. 刘佳雯, 娄秉盛, 李瑶, 等. Prevalence and risk factors of age-related macular degeneration in Uyghur residents aged 50 or above in southern Xinjiang region (新疆南疆地区 50 岁及以上维吾尔族人群年龄相关性黄斑变性患病率及危险因素) [J]. Chinese Journal of Experimental Ophthalmology (中华实验眼科杂志). 2019,37(1): 40-44.
AMD-28	Qiu-xia Bai, Ya-xing Wang, Dong-ning Chen. 白秋霞，王亚星，陈东宁. Population-based association between age-related macular degeneration and C-reactive protein in middle-aged and elderly people with physical examination* (中老年体检人群中老年黄斑变性与 C 反应蛋白的人群关联性研究) [J]. Chinese Journal for Clinicians (中国临床医生杂志). 2023,51(11):1315-1318.
AMD-29	Yan-ling Zhang, Li-juan Xing, Qi Jia, et al. 张艳玲, 邢丽娟, 贾琪, 等. Epidemiological Investigation of Eye Diseases Among Adults Aged 50 Years or Above of Futian A District (某区 50 岁及以上人群眼病及影响因素的分析) [J]. Guide of China Medicine (中国医药指南). 2020,18(21): 5-7.
AMD-30	Jie-ying Mai, Ming-hua Liao, Ting-ting Liao. 麦洁英，廖敏华，刘婷婷. A study of the prevalence of age-related macular degeneration* (老年性黄斑变性的患病状况研究) [J]. Laboratory Medicine and Clinic (检验医学与临床). 2017,14(04): 570-572.
AMD-31	Yu Guan, Mei Yang, Li-hua Kang, et al. 管宇, 杨梅, 康丽华, 等. Epidemiological survey of age-related macular degeneration in population aged≥50 years in Funing country, Jiangsu (江苏省阜宁县农村 50 岁及以上人群年龄相关性黄斑变性的流行病学调查) [J]. International Eye Science (国际眼科杂志). 2018,18(01): 133-136.
AMD-32	Hai-ming Xu, Xin Wang, Jing-wen Gong. 徐海铭, 王鑫, 龚静文. Epidemiological investigation of eye diseases among adults aged 50 years or older in the communities of Hangzhou in China (杭州市下城区 50 岁及以上人群眼病流行病学调查) [J]. Chinese Journal of Optometry Ophthalmology and Visual Science (中华眼视光学与视觉科学杂志). 2018,20(6): 333-338.

Study ID	Reference
AMD-33	Xiao-ying Zhang, Rui-juan Guan, Xin Yan, et al. 张晓英，关瑞娟，晏鑫，等. Risk factors analysis of age-related macular degeneration in Tibetan of plateau area and establishment of nomogram prediction model (高原地区藏族年龄相关性黄斑变性相关危险因素分析及诺莫预测模型建立) [J]. Chinese Journal of Experimental Ophthalmology (中华实验眼科杂志). 2022,40(03): 260-265.
AMD-34	Rui-Juan Guan, Ling Li, Xin Yan, et al. 关瑞娟，李凌，晏鑫，等. Analysis of ARMD related risk factors and establishment of Nomoto prediction model in high altitude area (高海拔地区 ARMD 相关危险因素分析及诺莫图预测模型的建立) [J]. International Eye Science (国际眼科杂志). 2020,20(12): 2139-2145.
AMD-35	Guang-ming Jin, Xiao-hu Ding, Wei Xiao, et al. Prevalence of age-related macular degeneration in rural southern China: the Yangxi Eye Study. The British journal of ophthalmology 2018;102:625-630.
AMD-36	Kai-yan Zhang, Qiong-lei Zhong, Si-ying Chen, et al. An epidemiological investigation of age-related macular degeneration in aged population in China: the Hainan study. Int Ophthalmol 2018;38:1659-1667.
AMD-37	Hui-juan Wang, Shan-zhen Liu, Li-na Sha, et al. 王慧娟，刘善珍，沙立娜，等. The analysis of the results of eye health examination in 1127 elderly people * (1127 例老年人眼健康检查结果分析) [J]. China Medical Devices (中国医疗设备). 2017,12:55-56.
AMD-38	Xi Cao, Zhong Xin, Shi-ming Li, et al. The Status of Maculopathy in Diabetes and Prediabetes Patients in a Population-Based Study Detected by Optical Coherence Tomography: The 2011 Health Examination Survey in Beijing. BioMed Research International 2017;6513076
AMD-39	Yan-hui Lin, Ting Peng, Ying Li, et al. The frequency of early age-related macular degeneration and its relationship with dietary pattern in Hunan, China: a cross-sectional study. BMC Ophthalmology 2022;22:324
AMD-40	Yao Li, Hua-yin Feng, Jing Fu, et al. 李瑶，冯华银，付竟，等. Risk Factors and Correlation Analysis of AMD in People Aged 50 and above in Xinjiang (新疆喀什市和库车地区 50 岁以上人群黄斑变性危险因素相关性分析) [J]. Chinese Medical Record (中国病案). 2023,24(2):57-61.

Notes: The Chinese publication list employed the journals' official English names or abbreviations, English titles were obtained from journals or literature databases (CNKI, Wanfang and CQVIP). Where official English translation of journal names is not available, a pinyin title is adopted; where the English translation of titles is not available, I translated the titles, labelled with "*" and marked as green.

Appendix 5. PRISMA 2020 reporting checklist

Section and Topic	Item #	Checklist item	Location where item is reported
**TITLE**			
Title	1	Identify the report as a systematic review.	Page 1
**ABSTRACT**			
Abstract	2	See the PRISMA 2020 for Abstracts checklist.	Page 1, 2
**INTRODUCTION**			
Rationale	3	Describe the rationale for the review in the context of existing knowledge.	Page 3
Objectives	4	Provide an explicit statement of the objective(s) or question(s) the review addresses.	Page 4
**METHODS**			
Eligibility criteria	5	Specify the inclusion and exclusion criteria for the review and how studies were grouped for the syntheses.	Page 4, 5
Information sources	6	Specify all databases, registers, websites, organisations, reference lists and other sources searched or consulted to identify studies. Specify the date when each source was last searched or consulted.	Page 4
Search strategy	7	Present the full search strategies for all databases, registers and websites, including any filters and limits used.	Page 4
Selection process	8	Specify the methods used to decide whether a study met the inclusion criteria of the review, including how many reviewers screened each record and each report retrieved, whether they worked independently, and if applicable, details of automation tools used in the process.	Page 4
Data collection process	9	Specify the methods used to collect data from reports, including how many reviewers collected data from each report, whether they worked independently, any processes for obtaining or confirming data from study investigators, and if applicable, details of automation tools used in the process.	Page 6
Data items	10a	List and define all outcomes for which data were sought. Specify whether all results that were compatible with each outcome domain in each study were sought (e.g. for all measures, time points, analyses), and if not, the methods used to decide which results to collect.	Page 6
	10b	List and define all other variables for which data were sought (e.g. participant and intervention characteristics, funding sources). Describe any assumptions made about any missing or unclear information.	Page 6
Study risk of bias assessment	11	Specify the methods used to assess risk of bias in the included studies, including details of the tool(s) used, how many reviewers assessed each study and whether they worked independently, and if applicable, details of automation tools used in the process.	Not applicable
Effect measures	12	Specify for each outcome the effect measure(s) (e.g. risk ratio, mean difference) used in the synthesis or presentation of results.	Page 6
Synthesis methods	13a	Describe the processes used to decide which studies were eligible for each synthesis (e.g. tabulating the study intervention characteristics and comparing against the planned groups for each synthesis (item #5)).	Page 7
	13b	Describe any methods required to prepare the data for presentation or synthesis, such as handling of missing summary statistics, or data conversions.	Page 7-8
	13c	Describe any methods used to tabulate or visually display results of individual studies and syntheses.	Page 7-10
	13d	Describe any methods used to synthesize results and provide a rationale for the choice(s). If meta-analysis was performed, describe the model(s), method(s) to identify the presence and extent of statistical heterogeneity, and software package(s) used.	Page 7-10
	13e	Describe any methods used to explore possible causes of heterogeneity among study results (e.g. subgroup analysis, meta-regression).	Page 8-10
	13f	Describe any sensitivity analyses conducted to assess robustness of the synthesized results.	Page 8
Reporting bias assessment	14	Describe any methods used to assess risk of bias due to missing results in a synthesis (arising from reporting biases).	Not applicable
Certainty assessment	15	Describe any methods used to assess certainty (or confidence) in the body of evidence for an outcome.	Not applicable

PRISMA 2020 Checklist

Section and Topic	Item #	Checklist item	Location where item is reported
**RESULTS**			
Study selection	16a	Describe the results of the search and selection process, from the number of records identified in the search to the number of studies included in the review, ideally using a flow diagram.	Page 10
	16b	Cite studies that might appear to meet the inclusion criteria, but which were excluded, and explain why they were excluded.	Page 10
Study characteristics	17	Cite each included study and present its characteristics.	Online supplementary document
Risk of bias in studies	18	Present assessments of risk of bias for each included study.	Not applicable
Results of individual studies	19	For all outcomes, present, for each study: (a) summary statistics for each group (where appropriate) and (b) an effect estimate and its precision (e.g. confidence/credible interval), ideally using structured tables or plots.	Page 10-13
Results of syntheses	20a	For each synthesis, briefly summarise the characteristics and risk of bias among contributing studies.	Not applicable
	20b	Present results of all statistical syntheses conducted. If meta-analysis was done, present for each the summary estimate and its precision (e.g. confidence/credible interval) and measures of statistical heterogeneity. If comparing groups, describe the direction of the effect.	Page 10-13
	20c	Present results of all investigations of possible causes of heterogeneity among study results.	Page 10-13
	20d	Present results of all sensitivity analyses conducted to assess the robustness of the synthesized results.	Page 11
Reporting biases	21	Present assessments of risk of bias due to missing results (arising from reporting biases) for each synthesis assessed.	Not applicable
Certainty of evidence	22	Present assessments of certainty (or confidence) in the body of evidence for each outcome assessed.	Not applicable
**DISCUSSION**			
Discussion	23a	Provide a general interpretation of the results in the context of other evidence.	Page 13-16
	23b	Discuss any limitations of the evidence included in the review.	Page 16
	23c	Discuss any limitations of the review processes used.	Page 16
	23d	Discuss implications of the results for practice, policy, and future research.	Page 17
**OTHER INFORMATION**			
Registration and protocol	24a	Provide registration information for the review, including register name and registration number, or state that the review was not registered.	Page 4
	24b	Indicate where the review protocol can be accessed, or state that a protocol was not prepared.	Not applicable
	24c	Describe and explain any amendments to information provided at registration or in the protocol.	Not applicable
Support	25	Describe sources of financial or non-financial support for the review, and the role of the funders or sponsors in the review.	Page 17
Competing interests	26	Declare any competing interests of review authors.	Page 17
Availability of data, code and other materials	27	Report which of the following are publicly available and where they can be found: template data collection forms; data extracted from included studies; data used for all analyses; analytic code; any other materials used in the review.	Page 17

From: Page MJ, McKenzie JE, Bossuyt PM, Boutron I, Hoffmann TC, Mulrow CD, et al. The PRISMA 2020 statement: an updated guideline for reporting systematic reviews. BMJ 2021;372:n71. doi: 10.1136/bmj.n71
